# In situ product removal via reactive extraction in itaconic acid fermentation with *Ustilago cynodontis*

**DOI:** 10.1186/s13068-026-02753-7

**Published:** 2026-03-27

**Authors:** Katharina Maria Saur, Luca Antonia Grebe, Lina Wilke, Fabian Roweda, Pia Ergezinger, Robert Kiefel, Jørgen Barsett Magnus, Andreas Jupke

**Affiliations:** 1 Fluid Process Engineering (AVT.FVT), RWTH Aachen University, Forckenbeckstr. 51, 52074 Aachen, North Rhine-Westfalia Germany; 2 Biochemical Engineering (AVT.BioVT), RWTH Aachen University, Forckenbeckstr. 51, 52074 Aachen, North Rhine-Westfalia Germany; 3 WSS Research Centre “catalaix”, Aachen, Germany; 4 Institute for Bio- and Geosciences (IBG-2), Forschungszentrum Jülich GmbH, Wilhelm-Johnen-Straße, 52428 Jülich, North Rhine-Westfalia Germany; 5 Research, Development & Innovation, Evonik Operations GmbH, Rodenbacher Chaussee 4, 63457 Hanau, Hesse Germany

**Keywords:** ISPR, In situ product removal, *Ustilago cynodontis*, Itaconic acid, Weak organic acid stress, Reactive extraction, Trioctylamine, TOA, 2-octanone, Biocompatible solvents

## Abstract

Bio-based carboxylic acids are key platform chemicals for a circular economy, offering sustainable alternatives to fossil-derived products. Yet, high substrate and processing costs, along with narrow profit margins, restrict industrial-scale bioproduction. *In situ* product removal (ISPR) holds the potential to increase productivity and yield in fermentations by circumventing product inhibition. Thus, it presents a promising process intensification measure to bridge the commercial gap between bio-based and petrochemical platform chemicals. One effective method for product recovery is reactive extraction with trioctylamine. In the present study, this method was applied to itaconic acid (ITA) fermentations with *Ustilago cynodontis*. First, the successful operation of dispersion-based apparatuses for reactive extraction was proven using small-scale mixer–settlers, with 1-octanol and the biocompatible 2-octanone as diluents. We then developed an improved feeding profile, lowering byproduct formation by 72 %. Subsequently, we demonstrated the feasibility of ISPR using a perfusion bioreactor with an external membrane coupled to reactive extraction and back-extraction. After 233 h of fermentation, the total amount of ITA produced was increased by 26 %, and productivity was 21 % higher compared to extended-batch fermentations. However, the yield was only slightly improved by 5 %. Ultimately, we identified product toxicity far below the maximum titer of 80 g $\hbox {L}^{-1}$ as a key bottleneck in ISPR fermentations with *U. cynodontis*. The results underscore the potential of ISPR and warrant further investigation in this field.

## Introduction

In the transition to a circular economy, bio-based carboxylic acids are promising platform chemicals [1]. With their potential to replace fossil-derived resources in the chemical industry, they can act as either drop-in solutions or novel materials [1–3]. However, low profit margins [4] in relation to high substrate costs [5–7], high additive consumption [6–8], and substantial energy requirements for downstream processing (DSP) [5, 8] hinder widespread introduction of these alternatives onto the market [4]. As a result, only a limited number of carboxylic acids, such as citric and lactic acid, are produced at large industrial scale through biological manufacturing routes [9, 10]. The present work aims to increase the commercial viability of other bio-based carboxylic acids by implementing process intensification measures, using itaconic acid (ITA) as a case study.

Biotechnological production of ITA dates back to the 1950s. Typically, fermentation is operated with *Aspergillus terreus* as production host, and ITA is purified by evaporation and multiple crystallization steps [11, 12]. The production process not only faces challenges regarding a high carbon footprint for evaporation [13, 14], but also needs extensive substrate pretreatment to prevent filamentous growth of *A. terreus* [11, 15–17]. Furthermore, there are regulatory hurdles arising from the classification of *A. terreus* as a biosafety level 2 organism [18]. Accordingly, ITA is currently only used in synthetic latex production, resulting in a small global production volume of approximately 40,000 t/a in 2018 [19]. However, ITA has the potential to serve as a versatile precursor for manufacturing polymers and hydrogels [20–24] and can act as a substitute for maleic acid anhydride in the synthesis of polyester resins [25–27]. To access these new applications, alternative production hosts, such as *Ustilago* sp., with a broad substrate spectrum and yeast-like growth profile, are investigated in the literature [28–36]. Especially *Ustilago cynodontis* has gained increased interest due to its low fermentation pH value of 3.6 [37], reducing acid and base consumption as well as saline waste production in DSP [5, 38]. In recent works, a production process similar to *A. terreus* has been established for *U. cynodontis* at estimated operational costs of 1.42 EUR per kg [5]. This is close to the current market price of ITA, which ranges from 1.08 EUR/kg to 1.82 EUR/kg, depending on location and market fluctuations [19, 39]. However, this mere adaptation of the *A. terreus* process to *U. cynodontis* is not enough to sufficiently lower the production costs and tap into new application possibilities for ITA. Looking at the fermentation key performance indicators (KPI) of *U. cynodontis*, it becomes clear that this new production organism is still inferior to *A. terreus*. *A. terreus* can achieve titers of up to 160 g $\hbox {L}^{-1}$ [16], a productivity of 1.15 $\hbox {g}_{\hbox {ITA}} \hbox {L}^{-1} \hbox {h}^{-1}$ [40] and near theoretical yield [41]. *U. cynodontis* shows feasible titers of 80 g $\hbox {L}^{-1}$ [37], a productivity of 0.59 $\hbox {g}_{\hbox {ITA}} \hbox {L}^{-1} \hbox {h}^{-1}$ and a maximum yield of 0.55 ${\hbox {g}}_{\hbox {ITA}} \hbox {g}_{\hbox {Glc}}^{-1}$ [38]. Most reported yields in literature, however, are between 0.38 ${\hbox {g}}_{\hbox {ITA}} \hbox {g}_{\hbox {Glc}}^{-1}$ and 0.48 ${\hbox {g}}_{\hbox {ITA}} \hbox {g}_{\hbox {Glc}}^{-1}$ due to high product inhibition [5, 37, 42]. As the carbon source accounts for the majority of production costs [5], an increase in production yield is vital. In combination with the concerns regarding the carbon footprint of the DSP based on multiple crystallization steps [13, 14], this emphasizes the need for new processes with not only high yields, but also reduced energy and additives consumption.

One process intensification measure to address some of these challenges is *in situ* product removal (ISPR). By directly coupling fermentation with DSP, ISPR lowers the product concentration in the fermentation broth during cultivation, thereby mitigating product inhibition [43]. Thus, compared to batch cultivations, ISPR allows for a prolonged production phase [44], and the production rate is often higher [45–48]. In some cases, ISPR can also improve the yield [49, 50]. In addition, the product concentration can be increased after the first capturing step [45, 46, 51–55] and the aqueous waste generated during the process can be reduced, as more product is produced per kg fermentation broth [56]. The literature describes various unit operations for ISPR, including distillation [57, 58], gas-stripping [49, 51], crystallization [59], electrodialysis [46], adsorption [42, 60], extraction [44, 50, 61, 62] and reactive extraction [55, 63]. For carboxylic acids, gas-stripping or distillation is often energy-intensive due to their high boiling points [56]. Crystallization would require low product solubility and a sufficiently low pH to achieve high protonated acid concentrations [59]. If the broth has a neutral pH, electrodialysis can be applied for direct acid removal [46, 53]. For fermentation at lower pH values producing highly soluble compounds, adsorption, extraction, or reactive extraction can be directly coupled to fermentation without pretreating the fermentation broth [42, 50, 55, 63]. For ITA production with *U. cynodontis*, the potential of ISPR by adsorption has already been highlighted by Pastoors et al. (2023) [42], showing an 8% increase in yield and an 11% boost in productivity. Building on the findings from Pastoors et al. [42], we will implement ISPR by reactive extraction, thereby expanding the toolbox available for process intensification with *U. cynodontis*. We will use the same production organism, fermentation protocol, and media as in previous works [37, 38, 42] to benchmark our results and discuss the influence of ISPR on fermentation KPI.

Reactive extraction is one of the most frequently mentioned separation techniques for carboxylic acid recovery in literature [56]. The addition of a complexation agent such as trioctylamine (TOA) [55, 64] or trioctylphosphine oxide (TOPO) [65–67] to an organic phase increases distribution coefficients, facilitating the isolation of polar compounds from highly diluted solutions [55, 68]. While the current literature is primarily concerned with organic phases consisting of complexation agents in long-chain alcohols or alkanes [56], there is the possibility to use a wide variety of diluent and extractant combinations [69, 70]. As a result, reactive extraction systems can potentially be customized to specific applications based on the extraction mechanism, distribution coefficient, and selectivity [56, 69, 70]. Furthermore, phase separation [71], and biocompatibility can be adjusted by selecting appropriate reactive extraction systems [55, 72, 73]. An additional feature, proprietary to tertiary amines, such as TOA, is their protonation at the liquid–liquid interface. It enables the extraction of not only the protonated acid, but also of its dissociated form [74–80]. Thus, reactive extraction with TOA is constrained by the protonation of TOA, rather than that of the carboxylic acid [79]. This characteristic enables efficient extraction over a broader pH range than adsorption [42, 55, 81], reactive extraction with phosphate-based adsorbents [66, 82, 83], or physical extraction [84–86], where only the protonated acid is removed from the fermentation broth [56]. Keeping a direct coupling to fermentation in mind, this could offer a wider pH-operating range and increased applicability without the need for pH adjustment for high extraction yields [55, 87].

Despite the promising increase in pH range and the flexibility of reactive extraction systems, mainly process concepts are published [88–90]. Only a few fermentations implementing ISPR by reactive extraction with a tertiary amine are reported in the literature [50, 55, 63]. This scarcity can be partially attributed to experimental complexity, and to challenges in phase separation and biocompatibility [50, 56, 63]. To overcome these challenges, we will establish a perfusion bioreactor with an external membrane system [46, 53]. Unlike the direct addition of an organic phase to the fermenter, commonly found in studies for both reactive [50, 63] and physical extraction [91–95], this setup will omit phase toxicity that arises from the direct contact of cells and the organic phase [96]. Only molecular phase toxicity [97], originating from the cross-dissolved organic solvent, will be present in our setup. Furthermore, the formation of Pickering emulsions [92, 94, 98] due to cells stabilizing the liquid–liquid interface, will be bypassed. This will enable a fermentation profile with high energy input and mass transfer rates [61].

For reactive extraction, mainly dispersion-free apparatuses are found in the literature due to challenges associated with impaired phase separation [55, 56]. However, for industrial applications, mixer–settlers or columns are easier to implement and exhibit higher mass transfer rates [99, 100]. Consequently, to evaluate the feasibility of reactive extraction of carboxylic acids from fermentation broth using dispersion-based apparatuses, we will implement mixer–settlers at lab-scale for reactive extraction. A subsequent back-extraction will be performed via a pH shift with NaOH to deplete and recycle the organic phase. We will use TOA-1-octanol, one of the most frequently mentioned reactive extraction systems in the literature [56], for the initial commissioning of mixer–settlers. Afterward, we will employ 2-octanone as a diluent to increase the biocompatibility of the solvent. The effects of diluent change on mixer–settler operation are discussed.

By aligning fermentation and purification, we obtain a suitable setup for ISPR by reactive extraction. A comparison to the literature on both fermentation [37, 38, 42] and reactive extraction [56] will reveal some of the main bottlenecks in ITA production with *U. cynodontis*, thereby determining further steps to improve the potential of ISPR as a process intensification method for this strain.

## Materials and methods

For reactive extraction, 92% TOA and all diluents except 2-octanone were purchased from Sigma Aldrich (St. Louis, Missoury, USA). 2-Octanone was acquired from Thermo Fischer Scientific (Waltham, Massachusettes, USA). For fermentation, most medium components were obtained from Carl Roth (Karlsruhe, Germany). Only MgSO_4_, FeSO_4_ and Antifoam 204 were purchased from Sigma Aldrich (St. Louis, Missoury, USA). The production strain *U. cynodontis* NBRC 9727 $\Delta fuz7$ $\Delta cyp3$ *P*$_{{etef}}$*mttA P*$_{{ria1}}$*ria1* was kindly provided by Prof. Nick Wierckx (Institute of Bio and Geosciences IBG-1: Biotechnology, Forschungszentrum Jülich, Jülich, Germany). The strain has been genetically modified to show low byproduct accumulation, increased ITA formation, and a yeast-like growth pattern [31, 37]. So far, erythritol has been identified as the main remaining byproduct [37].

### pH-dependent reactive extraction and back-extraction

To obtain pH-dependent equilibrium data on reactive extraction, 0.5 mol ${\hbox {L}^{-1}}$ ITA solutions were prepared using water saturated with the corresponding organic phase. Different pH values were adjusted with 2 mol ${\hbox {L}^{-1}}$ NaOH, which was also saturated with the organic phase and contained 0.5 mol ${\hbox {L}^{-1}}$ ITA. Thereby, a pH adjustment did not influence the ITA concentration in the solution. The organic phase consisted of 0.5 mol ${\hbox {L}^{-1}}$ TOA in the corresponding diluent and was saturated with water. Reactive extraction was performed at a volume phase ratio of $V_{\mathrm {org:aq}}=1:1$ and the samples were equilibrated laying horizontally in an orbital shaker with 5 mm shaking diameter at 100 rpm (Adolf Kühner AG, Birsfelden, Switzerland) for at least 4 h at room temperature. Phase separation was obtained by centrifugation with a Rotana 460R centrifuge (Andreas Hettich GmbH, Tuttlingen, Germany) at 4000 rpm for 10 min using a swing bucket rotor. The aqueous phase was analyzed by high-performance liquid chromatography (HPLC) and pH measurements. For back-extraction, the organic phase was first loaded by reactive extraction of 0.5 mol ${\hbox {L}^{-1}}$ ITA at pH 2.0 using a volume phase ratio of $V_{\mathrm {org:aq}}=1:1$. The loaded organic phase was then contacted with saturated NaOH at different concentrations of up to 2 mol ${\hbox {L}^{-1}}$. The volume phase ratio was also at $V_{\mathrm {org:aq}}=1:1$. The samples were equilibrated and analyzed as in reactive extraction.

### Reactive extraction and back-extraction in lab-scale mixer–settlers

For reactive extraction and back-extraction in ISPR experiments, a setup with two lab-scale mixer–settlers was used. The design of the lab-scale mixer–settlers was presented previously [101] and consisted of a 100 mL GL45 laboratory flask modified with a plane flange and connected to a DN25 glass tubing. For phase separation, a glass end cap with three GL14 openings and a plane flange was connected to the end of the glass tubing. The total volume of the mixer–settlers was determined to 253 mL and the settler length was 23 cm. Dispersion of the organic phase was achieved using a single disk blade stirrer with a diameter of 4 cm, connected to a Witeg Labortechnik GmbH overhead stirrer HS-30D (Wertheim, Germany). For back-extraction, a similar setup was used. The mixer–settlers were filled using Ismatec Reglo ICC four-channel digital peristaltic pumps with 8 rollers (Omnilab Laborzentrum, Bremen, Germany). For tubing, PTFE tubes and solvent-resistant peristaltic Tygone pump tubing were applied. The interface level was adjusted manually using hydrostatics with a U-pipe. The runtime required to reach steady state was determined to be 60 min (Appendix A.1). All phases were saturated with solvent and water experiments.

Prior to ISPR experiments, appropriate operating conditions were established for reactive extraction and back-extraction to ensure that extraction in the mixer–settler continued until equilibrium was reached and the rate of ITA removal was matched to the rate of ITA production during fermentation. Based on the desired fermentation conditions, we employed aqueous solutions with ITA concentrations ranging from 5 g $\hbox {L}^{-1}$ to 40 g $\hbox {L}^{-1}$, at flow rates of 5 mL ${\hbox {min}^{-1}}$ and 10 mL ${\hbox {min}^{-1}}$. The starting pH was set to 3.6 in all cases. For the organic phase, 0.5 mol ${\hbox {L}^{-1}}$ TOA in either 1-octanol or 2-octanone was applied. The feed volume phase ratio for reactive extraction was set to $V_{\mathrm {org:aq}}=1:1$, thereby avoiding limitations concerning TOA binding sites in the organic phase and ensuring proper mixing in the mixer–settler. The stirrer speed was within a range of 300 rpm to 600 rpm. To establish operating parameters for back-extraction, the organic phase was loaded by reactive extraction of 0.125 mol ${\hbox {L}^{-1}}$, 0.25 mol ${\hbox {L}^{-1}}$ and 0.5 mol ${\hbox {L}^{-1}}$ ITA solution at pH 2.0 using a volume phase ratio of $V_{\mathrm {org:aq}}=1:1$. For back-extraction, 0.5 mol ${\hbox {L}^{-1}}$ to 2 mol ${\hbox {L}^{-1}}$ NaOH at varying feed volume phase ratios were used. Stirrer speeds for back-extraction were selected similar to reactive extraction between 300 rpm to 600 rpm. To determine reactive extraction and back-extraction yield, the aqueous phase was analyzed by HPLC and pH measurements. If not indicated otherwise, the ITA concentration in the organic phase was calculated based on the ITA depletion of the aqueous phase (Analytics). In addition, equilibrium data was collected for every mixer–settler operating point using phase volume ratios corresponding to the mixer–settler experiments (Sect. pH-dependent reactive extraction and back-extraction).

### Reactive extraction performance evaluation

Despite saturation of both phases prior to the experiments, changes in aqueous phase and organic phase volumes were observed due to co-extraction of water [102, 103] (Appendix A.2). In equilibrium experiments, the volume changes were accounted for by measuring the volumes of both phases before and after reactive extraction and back-extraction. The yield for reactive extraction was calculated based on the ITA concentration in the aqueous phase before and after reactive extraction ($c_{\mathrm {ITA,aq,t_{0}}}$, $c_{\mathrm {ITA,aq,t_{1}}}$) and the corresponding phase volumes $V_{\mathrm {aq, t_0}}$ and $V_{\mathrm {aq, t_1}}$ (Eq. 1).

1 $$\begin{aligned} Y_{\textrm{REx}} = 1 -\frac{c_{\mathrm {ITA, aq, t_1}}\cdot V_{\mathrm {aq, t_1}}}{c_{\mathrm {ITA, aq, t_0}}\cdot V_{\mathrm {aq,t_0}}} \end{aligned}$$

The ITA concentration in the organic phase was calculated based on the organic phase volume after reactive extraction ($V_\mathrm {org,t_1}$), the initial mass of ITA ($m_{\mathrm {ITA,aq,t_0}}$) and $Y_{\textrm{REx}}$ (Eq. 2).

2 $$\begin{aligned} c_{\mathrm {ITA,org,t_1}} = \frac{m_{\mathrm {ITA,aq,t_0}}\cdot Y_{\textrm{REx}}}{V_\mathrm {org,t_1}} \end{aligned}$$

Using $c_{\mathrm {ITA,org, t_1}}$, the back-extraction yield was determined with the volume of the organic phase used for back-extraction $(V_\mathrm {org,t_1}^*)$, the volume of the aqueous phase after back-extraction $(V_\mathrm {NaOH, t_2})$, and the ITA concentration in the aqueous phase after back-extraction $(c_\mathrm {ITA,NaOH,t_2})$ (Eq. 3).

3 $$\begin{aligned} Y_{\mathrm{BEx}} = \frac{c_\mathrm {ITA,NaOH,t_2}\cdot V_\mathrm {NaOH, t_2}}{c_\mathrm {ITA,org,t_1}\cdot V_\mathrm {org,t_1}^*} \end{aligned}$$

In mixer–settler experiments, the phase volumes were not only influenced by co-extraction of water, but also by the manually regulated position of the liquid–liquid interface. Depending on the sample time, a predominant flow of either the organic or aqueous phase into the sample containers could have occurred. Therefore, the yield for reactive extraction and back-extraction in mixer–settler experiments was solely calculated based on the concentration difference of ITA in the corresponding aqueous and organic phases. However, since the mixer–settler experiments were performed with low loadings, the relative concentration change of the aqueous phase was below 3 % for reactive extraction and below 6 % for back-extraction (Appendix A.2), and therefore, only slight deviations from the actual values were expected.

To match fermentation productivity and ITA removal by reactive extraction in mixer–settler experiments, we calculated the average ITA removal rate $R_{\textrm{REx,24h}}$ based on the desired 6 h separation intervals every 24 h. As a basis for calculations, we used the total amount of ITA removed by reactive extraction ($m_{\textrm{ITA,REx}}$) obtained from the total volume of aqueous phase fed through reactive extraction ($V_{\textrm{aq,REx}}$), as well as $c_{\mathrm {ITA,aq,t_{0}}}$ and $Y_{\textrm{REx}}$ (Eqs. 4 and 5).

4 $$\begin{aligned} & m_{\textrm{ITA,REx}} = V_{\textrm{aq,REx}}(t) \cdot c_\mathrm {ITA, aq, t_0} \cdot Y_{\textrm{REx}} \end{aligned}$$

5 $$\begin{aligned} & R_{\textrm{REx,24h}} =\frac{m_{\textrm{ITA,REx}}}{t} \cdot \frac{6h}{24h} \end{aligned}$$

### Shake flask cultivations

Shake flask cultivations of *U. cynodontis* were performed in 250 mL shake flasks with 10 mL of modified Verduyn medium as described by Pastoors et al. (2023) [42]. The medium was supplemented with a vitamin solution based on the medium from Tehrani et al. (2019) [37]. Correspondingly, the medium for shake flask cultivations consisted of 50 g $\hbox {L}^{-1}$ glucose, 4 g $\hbox {L}^{-1}$ NH_4_Cl, 2 g $\hbox {L}^{-1}$ KH_2_PO_4_, 0.4 g $\hbox {L}^{-1}$ MgSO_4_$\cdot$7H_2_O, 0.01 g $\hbox {L}^{-1}$ FeSO_4_$\cdot$7H_2_O, 19.25 g $\hbox {L}^{-1}$ (0.1 mol ${\hbox {L}^{-1}}$) MES buffer, 1 mL trace element solution and 1 mL vitamin solution. For all compounds mentioned above, stock solutions were prepared and combined accordingly. The pH of the KH_2_PO_4_ solution was adjusted to 6.0, and the pH of the MES buffer was set to 6.5 using NaOH. For the FeSO_4_ stock, the pH was set below 2 by adding 5 mol ${\hbox {L}^{-1}}$ H_2_SO_4_. To obtain the trace element solution, 15 g $\hbox {L}^{-1}$ EDTA, 3 g $\hbox {L}^{-1}$ FeSO_4_$\cdot$7H_2_O, 0.84 g $\hbox {L}^{-1}$ MnCl_2_$\cdot$2H_2_O, 4.5 g $\hbox {L}^{-1}$ ZnSO_4_$\cdot$7H_2_O, 0.3 g $\hbox {L}^{-1}$ CuSO_4_$\cdot$5H_2_O, 0.3 g $\hbox {L}^{-1}$ CoCl_2_$\cdot$6H_2_O, 0.4 g $\hbox {L}^{-1}$ Na_2_MoO_4_$\cdot$2H_2_O, 4.5 g $\hbox {L}^{-1}$ CaCl_2_$\cdot$2H_2_O, 1 g $\hbox {L}^{-1}$ H_3_BO_3_ and 0.1 g $\hbox {L}^{-1}$ KI were combined [42]. The vitamin solution comprised 0.05 g $\hbox {L}^{-1}$ D-biotin, 1 g $\hbox {L}^{-1}$ D-calcium pantothenate, 1 g $\hbox {L}^{-1}$ nicotic acid, 25 g $\hbox {L}^{-1}$ *myo*-inositol, 1 g $\hbox {L}^{-1}$ thiamine hydrochloride, 1 g $\hbox {L}^{-1}$ pyridoxine hydrochloride and 0.2 g $\hbox {L}^{-1}$ para-aminobenzoic acid. The medium was sterilized by filtration through a 0.2 $\upmu$m Millipore Steritop vacuum bottle filter (Merck Millipore, Billerica, Massachusetts, USA). Inoculation was conducted to reach an OD_600_ of 0.1 at a wavelength of 600 nm (OD_600_) from cryopreserved culture stocks. The culture stocks were prepared with 300 g $\hbox {L}^{-1}$ glycerol and stored at −80 $^{\circ }\text {C}$. Cultivation was performed at 30 $^{\circ }\text {C}$ and 350 rpm in a Climo-Shaker ISF1-X (Kühner AG, Birsfelden, Switzerland) with a shaking diameter of 50 mm.

### Biocompatibility testing of solvents

The biocompatibility of 0.5 mol ${\hbox {L}^{-1}}$ TOA in alcohol, ester, and ketone diluents was tested with nitrogen-limited cells in a screening medium similar to the shake flask medium (Sect. Shake flask cultivation), but with 80 g $\hbox {L}^{-1}$ glucose and without NH_4_Cl. Furthermore, MES buffer was replaced with a 0.1 mol ${\hbox {L}^{-1}}$ sodium citrate buffer to yield a medium pH of 4.0. The pH dropped slightly during screening due to ITA production, leading to a similar pH as during the production phase in fermentation [37]. To screen for the biocompatibility of different solvents, the screening medium was saturated with organic solvent systems. If not indicated otherwise, the organic solvent systems consisted of 0.5 mol ${\hbox {L}^{-1}}$ TOA in the corresponding diluent. The screening medium and the organic solvent systems were equilibrated overnight at room temperature in an orbital shaker with a 5 mm shaking diameter (Adolf Kühner AG, Birsfelden, Switzerland) at 35 rpm. Thereby, we obtained similar solvent concentrations as in extraction outside of the perfusion bioreactor, also at room temperature. Phases were separated by centrifugation in a Rotana 460R centrifuge (Andreas Hettich GmbH, Tuttlingen, Germany) at 4000 rpm for 10 min. Then the saturated media were transferred to a $\upmu$TOM device, enabling the measurement of the OTR in a 96-well plate (riplate RW, 2.0 mL round deepwell plate, HJ-BIOANALYTIK GmbH, Erkelenz, Germany) [104, 105]. To obtain sterile conditions, the 96-well plates were covered with gas-permeable sealing films (Aer-aSeal$^{\hbox {TM}}$, Sigma-Aldrich, St. Louis, USA). Operation conditions for OTR measurement were set as described by Dinger et al. (2022) [104]. The filling volume was at 300 $\upmu$L, incubation was conducted at 30 $^{\circ }\text {C}$ at 1000 rpm and a shaking diameter of 3 mm using a Climo-Shaker ISF1-X (Kühner AG, Birsfelden, Switzerland). The precultures in shake flasks were monitored by an in-house RAMOS device [104] harvested in the exponential phase [105, 106]. The wells were inoculated to reach an OD_600_ of 10. Experiments were performed in triplicate.

### Diluent stability toward NaOH in back-extraction

To determine the stability of diluents with an ester group in back-extraction, we equilibrated a fully water saturated organic phase with NaOH at concentrations ranging from 0.25 mol ${\hbox {L}^{-1}}$ to 2 mol ${\hbox {L}^{-1}}$. Equilibration was performed at a volume phase ratio of $V_{\mathrm {org:aq}}=1:1$ for 4 h in an orbital shaker with 5 mm shaking diameter at 100 rpm (Adolf Kühner AG, Birsfelden, Switzerland). Similar to reactive extraction and back-extraction experiments, the phases were separated by centrifugation at 4000 rpm for 10 min. The hydrolysis reaction was stopped by a fivefold dilution with 25 mmol ${\hbox {L}^{-1}}$ H_2_SO_4_ and the samples were analyzed by HPLC for the hydrolysis product acetic acid. All extraction experiments were performed at room temperature.

### Extended-batch fermentations

Fermentations of *U. cynodontis* have been established ranging from a 0.5 L to 105 L [5, 37] scale, employing different feeding strategies, pH profiles, power input, and biomass concentrations [5, 37, 38, 42, 105]. Since ISPR by adsorption has been published previously for cultivations by Pastoors et al. [42] with *U. cynodontis*, using an initial filling volume of 1.25 L, we followed this approach to establish our fermentation procedure at an initial volume of 3.5 L. This allowed us to obtain highly comparable data.

Fermentations were performed with a 5 L Sartorius Stedim Biotech GmbH (Göttingen, Germany) Biostat B fermenter. The medium was prepared similar to the shake flask medium (Sect. Shake Flask Cultivation), but with lower glucose concentrations of 20 g $\hbox {L}^{-1}$ and without MES buffer [42].

The dissolved oxygen tension (DOT) was measured using a VisiFerm DO sensor (Hamilton Bonaduz AG, Bonaduz, Switzerland) and set to 30% by adjusting the stirrer speed. Two six-blade disk impellers with a diameter of 6.4 cm were used for stirring. The initial stirring rate was set to 300 rpm based on the volumetric power input averaged from fermentations with starting volumes of 1.25 L and 105 L [5, 42]. Aeration was set to 3.5 Lmin$^{-1}$, corresponding to 1 vvm of the initial cultivation volume [5, 37, 105]. The temperature was controlled at 30 $^{\circ }\text {C}$ [42].

The pH was measured by an EasyFerm Plus 325 pH sensor (Hamilton Bonaduz AG, Bonaduz, Switzerland). The optimal pH value for ITA production in extended-batch cultivations is 3.6 [37, 38]. The initial pH value during the growth phase, however, varies among studies, even though its effect on fermentation KPI is considered minor [37]. Hosseinpour-Tehrani et al. (2019) [37] primarily worked with a continuous pH value of 3.6, while Ernst et al. (2024) [38] operated the growth phase fully at pH 6.5 before letting the pH drop to 3.6 for production. The method from Pastoors et al. (2023) [42], applied in this study, is located in between. The initial pH value was set to 6.5 and allowed to drop naturally by consumption of NH_3_ before being controlled with 10 mol ${\hbox {L}^{-1}}$ NaOH at pH 3.6.

The literature also contains different feeding strategies, ranging from pulsed-fed batch fermentations [37] over extended-batch fermentations with a set feed [38, 105], to feed profiles controlled by a glucose sensor [37]. In the work of Hosseinpour-Tehrani et al. (2019) [37], a switch from a pulsed extended-batch fermentation to a glucose concentration controlled at 20 g $\hbox {L}^{-1}$ improved the yield from 0.39 ${\hbox {g}}_{\hbox {ITA}} \hbox {g}_{\hbox {Glc}}^{-1}$ to 0.45 ${\hbox {g}}_{\hbox {ITA}} \hbox {g}_{\hbox {Glc}}^{-1}$ as less erythritol was formed as a byproduct. This is also reflected in the data by Pastoors et al. (2023) [42], where sequentially decreasing glucose feed rates were used to maintain substrate concentrations below 20 g $\hbox {L}^{-1}$. Therefore, to obtain a low byproduct formation, glucose was monitored by an enzymatic BioPat (R) Trace online measurement, provided by Sartorius Stedim Biotech GmbH (Göttingen, Germany), and controlled at 20 g $\hbox {L}^{-1}$. The glucose concentration in the feeding solution was 500 g $\hbox {L}^{-1}$.

OTR and carbon dioxide transfer rate (CTR), as well as respiratory quotient (RQ), were determined based on data from a BioPAT$\circledR$ Xgas - Online Off-Gas Analysis (Satorius Stedim Biotech GmbH, Göttingen, Germany). The mass of fed glucose and titrated base was tracked based on offline gravimetric measurements of the containers. By correlating the gravimetric measurements to the online measured pump rates and considering the density of both glucose feed and base, we obtained online measurements for the volume flow of glucose and base. Similarly, the fermenter was placed on a scale, and the filling volume was calculated based on offline density measurements. Fermentation monitoring and data collection were conducted with Sartorius BioPAT MFCS software from 2022 (Sartorius Stedim Biotech GmbH, Göttingen, Germany).

Inoculation was performed at an OD_600_ of 0.1 with precultures from shake-flask cultivation harvested after 28 h to 30 h during exponential growth. Samples were taken regularly and analyzed by HPLC. Furthermore, offline pH, density, OD_600_, and cell dry weight (CDW) were determined (Analytics).

### Membrane commissioning

For cell retention, a seven-channel ceramic hollow fiber membrane from atech innovations GmbH (Gladbeck, Germany) with a pore size of 0.21 $\upmu$m, a channel diameter of 6 mm and a length of 1 m, resulting in a filtration area of 0.13 $\hbox {m}^{2}$ was used. It was connected as an external loop and operated in co-current crossflow mode by a PureFlo 21 membrane pump (Xylem Water Solutions Deutschland GmbH, Washington DC, USA). The PureFlo 21 membrane pump was attached to an MS2-632-4, 0.18 kW, 4 pole, B34 motor with an integrated Smart Drive frequency inverter (JS Technik GmbH, Großenkneten, Germany). The primary objective was to develop a filtration module that was easy to operate and had a low tendency for fouling. For this initial proof-of-concept, the transmembrane pressure was minimized by maintaining an unobstructed flow through the retentate valve [107–109]. Thus, the static pressure on the retentate side was primarily controlled by the feed flow rate of the fermentation broth. The membrane was commissioned using fermentation broth from an extended-batch cultivation. The broth was stirred at 300 rpm and aeration was set to 3.5 L min$^{-1}$. Feed flow rates from 1.5 L ${\hbox {min}^{-1}}$ to 5 L ${\hbox {min}^{-1}}$ were tested. As the feed flow rate determined the residence time and thus the possibility of oxygen limitation, the maximum possible oxygen uptake rate ($OUR_{\textrm{max}}$) was calculated based on the feed flow rate ($\dot{V}_{\textrm{feed}}$), the maximum oxygen solubility at 30 $^{\circ }\text {C}$ ($c_{\mathrm {O_2, max}}$), the DOT and the 0.6 L bypass volume ($V_{\textrm{BP}}$) (Eq. 6).

6 $$\begin{aligned} OUR_{\textrm{max}} = \frac{\dot{V}_{\textrm{feed}}\cdot c_{\mathrm {O_2, max}} \cdot \frac{DOT}{100}}{V_{\textrm{BP}}} \end{aligned}$$

### ISPR fermentation in a perfusion bioreactor

**Fig. 1:**
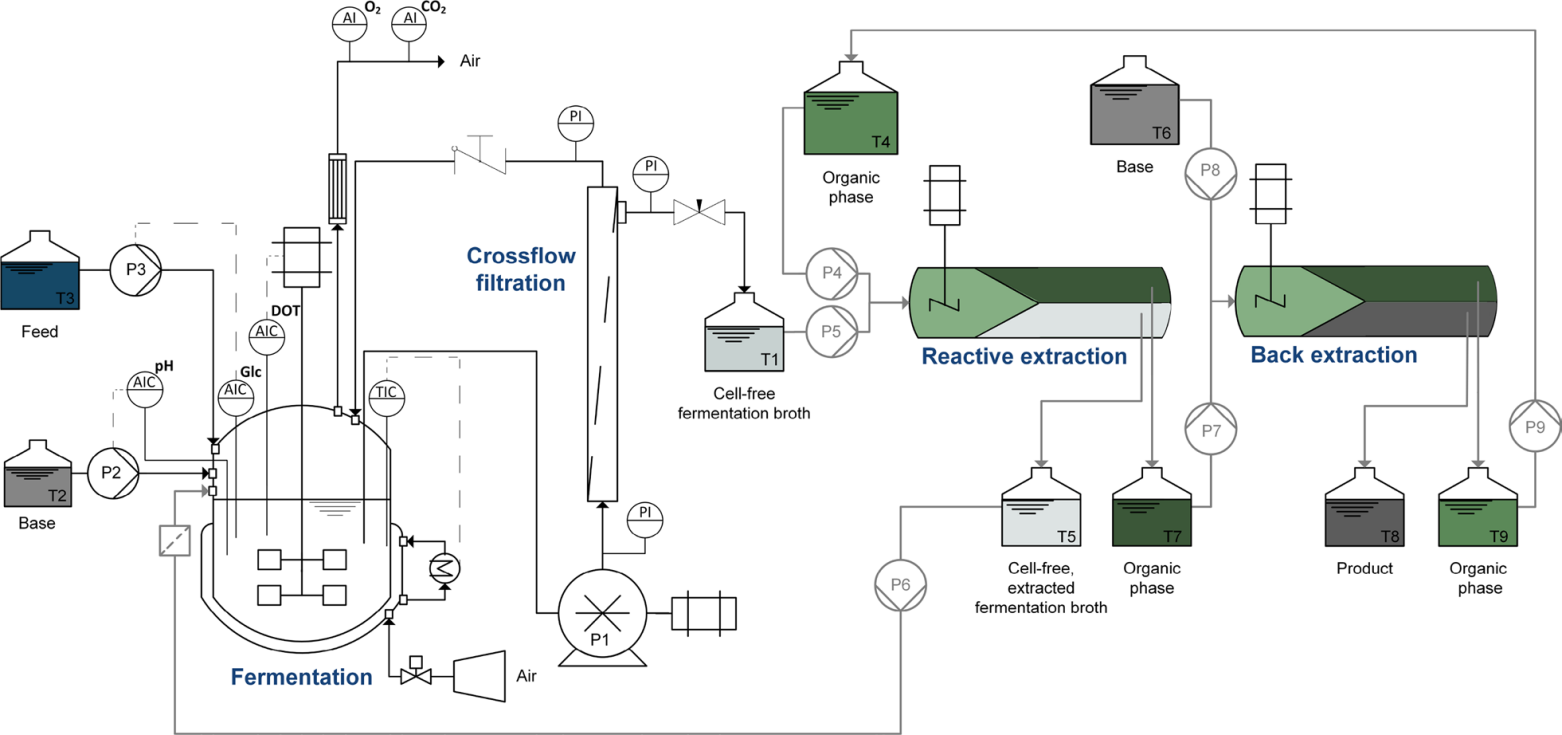
Detailed flow-sheet for ISPR fermentations. The black flow-path displays the operation of a perfusion bioreactor with an external membrane. The gray flow-path shows the setup used for ISPR

For ISPR fermentations, the fermentation unit (Extended-batch fermentations) was coupled to the external membrane loop (Membrane comissioning), and the permeate outlet was connected to the mixer–settler unit for reactive extraction and back-extraction (Sect. Reactive extraction and back-extraction in lab-scale mixer-settlers). The full setup is shown in Fig. 1.

Preculture and fermentation were conducted as described in Sects. Shake flask cultivations and Extended-batch Fermentations. The membrane was sterilized using 0.5 mol ${\hbox {L}^{-1}}$ NaOH cycling overnight on the retentate side and flushing with sterile water. After 72 h of fermentation, the fermenter was connected to the external loop via silicone tubing and the cells were cycled through the loop at a flow rate of 4.5 L min$^{-1}$. The permeate side of the membrane was opened using a manually operated needle valve after 88 h of cultivation. The emerging cell-free fermentation broth was fed to a buffer vessel. From there, 10 mL ${\hbox {min}^{-1}}$ were pumped to the mixer–settler for reactive extraction with equal volumes of 0.5 mol ${\hbox {L}^{-1}}$ TOA in 2-octanone saturated with water. Reactive extraction was conducted at a stirrer speed of 600 rpm and the depleted fermentation broth was recycled back into fermentation, passing through a dead-end 0.2 $\upmu$m sterile filter. The organic phase was continuously recovered in a second mixer–settler using 2 mol ${\hbox {L}^{-1}}$ NaOH at a feed phase volume ratio of $V_{\mathrm {org:aq}}=2:1$. The stirrer speed was also set to 600 rpm. The product solution was collected, and the organic phase was recycled back to reactive extraction. Samples were taken at regular intervals from the fermenter, the depleted aqueous phase after reactive extraction and the product phase. Sample analysis for fermentation and extraction samples was conducted as described in Sects.Extended-batch Fermentations and Analytics, respectively. Given that the mixer–settler construction did not permit unattended extraction, the operation time for ISPR runs was set to 6 h in 24 h intervals. Afterward, the manually operated needle valve at the permeate side of the membrane was closed, and fermentation was continued until the next extraction interval. In total, 5 extraction intervals were conducted. Afterward, the fermentation was continued with the membrane loop still in operation.

### Fermentation performance evaluation

Yield, space–time yield, productivity,CTR and OTR were calculated based on mass balances of each compound. Equation 7 describes the quantity of generated product P in extended-batch and ISPR cultivations considering the product in the fermenter ($m_{\textrm{P,F}}$), the product removed by fermenter sampling ($m_{\textrm{P,FS}}$), the product recovered by reactive extraction ($m_{\textrm{P, REx}}$) and the product removed from the product solution after reactive extraction due to sampling ($m_{\textrm{P, RExS}}$). $N_{1}$ and $N_{2}$ represent the total number of samples taken from fermentation and reactive extraction, respectively.

7 $$\begin{aligned} m_{\textrm{P}}(t) = m_{\textrm{P,F}}(t) + \displaystyle \sum _{i}^{N_{1}}m_{\textrm{P, FS},i} +m_{\textrm{P, REx}}(t) + \displaystyle \sum _{i}^{N_{2}}m_{\textrm{P, RExS},i} \end{aligned}$$

To determine the amount of glucose consumed, the glucose in the fermenter ($m_{\textrm{Glc, F}}$), the glucose in the fermentation medium at the beginning ($m_{\mathrm {Glc,t_0}}$), the glucose fed ($m_{\textrm{Glc,feed}}$), and the glucose removed by sampling during fermentation ($m_{\textrm{Glc, FS}}$) and reactive extraction ($m_{\textrm{Glc, RExS}}$) were taken into account. As glucose was not extracted into the organic phase (Appendix A.3), its removal from the product solution was not considered.

8 $$\begin{aligned} m_{\textrm{Glc, used}}(t) = m_{\mathrm {Glc,t_0}} + m_{\textrm{Glc,feed}}(t) - m_{\textrm{Glc,F}}(t) - \displaystyle \sum _{i}^{N_{1}}m_{\textrm{Glc, FS},i} -\displaystyle \sum _{i}^{N_{2}}m_{\textrm{Glc, RExS},i} \end{aligned}$$

Yield *Y*(*t*) in ${\hbox {g}}_{\hbox {ITA}} \hbox {g}_{\hbox {Glc}}^{-1}$ was calculated by dividing the total mass of ITA produced by the total mass of glucose used (Eq. (9)).

9 $$\begin{aligned} Y (t) = \frac{m_{\textrm{ITA}}(t)}{m_{\textrm{Glc, used}}(t)} \end{aligned}$$

STY in $\hbox {g}_{\hbox {ITA}} \hbox {L}^{-1} \hbox {h}^{-1}$ was determined by dividing the total mass of ITA produced by the fermenter volume ($V_{\textrm{F}}$), obtained from fermenter weight and density calculations after each time interval, and the fermentation time *t* (Eq. (10)).

10 $$\begin{aligned} STY (t) = \frac{m_{\textrm{ITA}}(t)}{V_{\textrm{F}}(t) \cdot t} \end{aligned}$$

OTR and CTR were calculated based on the gas mass balances, using $V_{\textrm{F}}$, the gas flow rate into the fermenter ($\dot{V}_{\textrm{A}}$), the molar volume of an ideal gas at 25 $^{\circ }\text {C}$ ($V_{\textrm{m}}$), the O_2_ and CO_2_ concentrations at the gas inlet of fermentation ($y_{\mathrm {O_2, in}}$ and $y_{\mathrm {CO_2, in}}$), and the concentrations in the exhaust gas ($y_{\mathrm {O_2, out}}$ and $y_{\mathrm {CO_2, out}}$). Details to the derivation of the mass balance-based approach resulting in Eqs. 11 and 12 can be found in Appendix A.3. Equations 11 and 12 solely quantify the amount of gas transferred into and out of the fermentation broth. They do not distinguish between physical dissolution of O_2_ and CO_2_ in the broth and the metabolic activity of the organism. However, the DOT is regulated to 30 %. Thereby, the concentration changes in the broth are close to zero and Eqs. 11 and 12 allow to draw conclusions on the metabolic state of the organisms, reflected by the OUR and the carbon dioxide evolution rate (CER). While this approach has been presented in previous works [5, 42], steady state might not always be given and slight deviations might occur. These limitations will be discussed where applicable.

11 $$\begin{aligned} & OUR =OTR= \frac{\dot{V}_{\mathrm{A}}}{V_{\textrm{F}}\cdot V_{\mathrm{m}}}\cdot (y_{\mathrm {O_2,in}}-\frac{1-y_{\mathrm {O_2,in}}-y_{\mathrm {CO_2,in}}}{1-y_{\mathrm {O_2,out}}-y_{\mathrm {CO_2,out}}}\cdot y_{\mathrm {O_2,out}}) \end{aligned}$$

12 $$\begin{aligned} & CER =CTR = \frac{\dot{V}_\mathrm{A}}{V_{\mathrm{F}}\cdot V_{\mathrm{m}}}\cdot (y_{\mathrm {CO_2,out}}\cdot \frac{1-y_{\mathrm {O_2,in}}-y_{\mathrm {CO_2,in}}}{1-y_{\mathrm {O_2,out}}-y_{\mathrm {CO_2,out}}}- y_{\mathrm {CO_2,in}}) \end{aligned}$$

From Eqs. 12 and 11, the RQ can be calculated as shown in Eq. 13. As described in detail in Saur et al. (2023) [5], the RQ of growth on pure glucose is at 1.01, and the RQ for ITA production from glucose is at 0.67.

13 $$\begin{aligned} RQ = \frac{CTR}{OTR} \end{aligned}$$

The amount of glucose going into maintenance during the production phase is determined based the CO_2_ balance (Eq. 14). CO_2_ originating from ITA production ($n_{\mathrm {CO_2, ITA}}$, 1 mol CO_2_ per mol ITA) and erythritol production ($n_{\mathrm {CO_2, Ery}}$, 2 mol CO_2_ per mol erythritol) is deduced from the total amount of CO_2_ produced ($n_{\textrm{CO2, tot}}$), yielding the amount of CO_2_ not accounted for ($n_{\mathrm {CO_2, m}}$). Assuming a full combustion of glucose into six molecules of CO_2_, the total amount of glucose going into maintenance ($n_{\textrm{Glc, m}}$) can be determined as described in Eq. 15.

14 $$\begin{aligned} & n_{\mathrm {CO_2, m}}(t) = n_{\mathrm {CO_2, tot}}(t) - n_{\mathrm {CO_2, ITA}}(t) - n_{\mathrm {CO_2, Ery}}(t) \end{aligned}$$

15 $$\begin{aligned} & n_{\textrm{Glc, m}}(t) = \frac{n_{\mathrm {CO_2, m}}(t)}{6} \end{aligned}$$

When maintenance is calculated for each fermentation sample point, data from after membrane implementation or after a reactive extraction interval is omitted. Instead, the data is averaged over 24 h to the next sample point to yield consistent data and to reflect the full time interval.

For carbon balancing, we considered the total glucose consumed (Eq. 8 in correlation with the glucose used for ITA, erythritol, and mannitol (Eq. 7), as well as for maintenance (Eq. 14 and 15) and for biomass formation. We assumed a biomass composition as published by Klement et al. (2012) [110] for *U. maydis* under nitrogen-limited conditions, which closely resembles the composition published by Liebal et al. (2022) [111].

### Analytics

HPLC analysis was performed in triplicate with an Agilent 1260 Infinity II (Agilent Scientific Instruments, Santa Clara, USA) setup, containing a G7112B binary pump, G7167A multisampler, and a G7116A column compartment. 5 $\upmu$L samples were injected and analyzed with a carboxylic acid resin column of 8 mm in diameter and 10 cm in length (CS Chromatography Service GmbH, Langerwehe, Germany). 2.5 mmol ${\hbox {L}^{-1}}$ H_2_SO_4_ was used as eluent at a flow rate of 1 mL ${\hbox {min}^{-1}}$. The temperature for analysis was set to 30 $^{\circ }\text {C}$. For detection and quantification, a refractive index detector G7162A (Agilent Scientific Instruments, Santa Clara, USA) was operated at 30 $^{\circ }\text {C}$. Evaluation was performed using Open Lab Software 3.4.5 (Agilent Scientific Instruments, Santa Clara, USA). Anion exchange chromatography was conducted using a 930 Compact IC Flex (Metrohm, Filderstadt, Germany) with Metrosep A Supp 7 - 250/4.0 column. Injection volume was set to 20 $\upmu$L. The flow rate of the eluent, containing 3.6 mmol ${\hbox {L}^{-1}}$ Na_2_CO^3^, was set to 0.7 mL min^-1^ and for detection, the built-in conductivity measurements were used.

pH was measured at room temperature with a SevenCompact pH S220-Basic pH-meter (Mettler Toledo, Columbus, USA) and an InLab^®^ Micro pH electrode (Mettler Toledo, Columbus, USA). Density measurements were conducted with DMA35 basic density meter (Anton Paar, Graz, Austria) at room temperature. OD_600_ was measured using a Fisherbrand^TM^ Cell Density Meter (Fischer Scientific GmbH, Waltham, USA) at a wavelength of 600 nm.

For CDW, 2 mL of broth was centrifuged at 12,000 rpm for 10 min using a 5810 R centrifuge (Eppendorf SE, Hamburg, Germany) in a 2 mL reaction vial. The supernatant was removed, and the pellet was dried at 40 $^{\circ }\text {C}$ at 200 mPa sec in a Vacutherm VT 6060 M oven (Fischer Scientific GmbH, Waltham, USA) and quantified gravimetrically.

Microscopic images were recorded with an Eclipse E600 microscope (Nikon, Tokyo, Japan) at 1000$\times$ magnification.

## Results and discussion

Initially, we will setup mixer–settlers for reactive and back-extraction. Subsequently, the rationale for selecting the 2-octanone-based solvent ibes validated. After implementing a suitable feeding profile, a fermentation with ISPR by reactive extraction will be conducted. Finally, the fermentation KPI will be discussed in relation to previous data from extended-batch cultivations [37] and ISPR fermentations by adsorption [42] to identify bottlenecks in ITA production with *U. cynodontis*.

### pH-dependent extraction of ITA with TOA

**Fig. 2:**
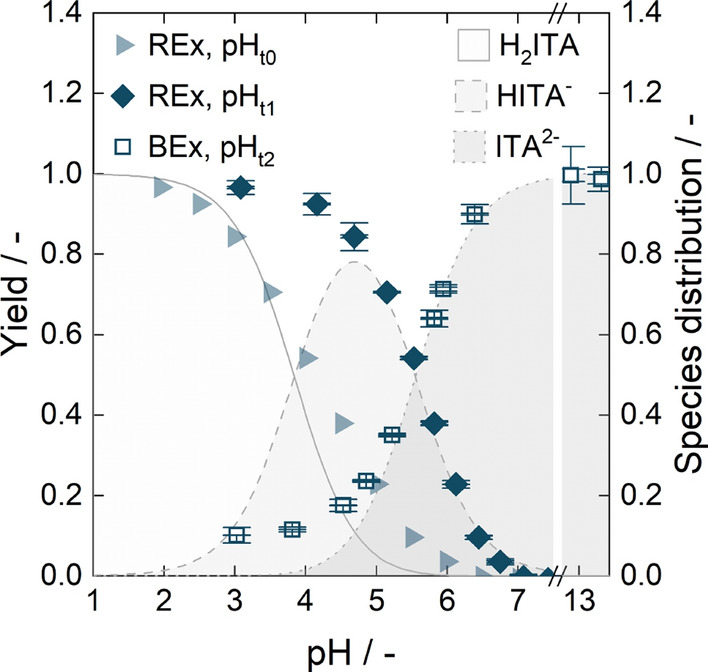
Equilibrium data for pH-dependent extraction of ITA by TOA in 1-octanol. The yield is displayed depending on starting pH (REx, ${{\textrm{pH}}_{{t}_{0}}}$) and on equilibrium pH (REx, ${{\textrm{pH}}_{{t}_{1}}}$), illustrating the pH shift due to ITA extraction. For back-extraction with NaOH, only the equilibrium pH is shown (BEx, ${{\textrm{pH}}_{{t}_{2}}}$)

To illustrate the reactive extraction mechanism with TOA, we investigated equilibrium data for reactive extraction of 0.5 mol ${\hbox {L}^{-1}}$ ITA at different pH values using 0.5 mol ${\hbox {L}^{-1}}$ TOA in 1-octanol. During extraction, the pH of the aqueous solution increased as the carboxylic acid was extracted. To illustrate this effect, the yield is depicted depending on starting and equilibrium pH after reactive extraction (Fig. 2). At a starting pH of 2.0, a maximum yield of 96.6 ± 0.2% was reached. As the initial pH increased, the yield decreased. For the fermentation pH of 3.6, the yield was below 70 %. The species distribution of ITA indicated by the shaded background illustrates extraction of both fully protonated ITA (H_2_ITA) by unprotonated TOA and once deprotonated ITA (HITA^-^) by protonated TOA. The extraction of fully deprotonated ITA (ITA^2-^) could not be confirmed, as the protonation of TOA at the liquid–liquid interface, determining extraction efficiency [74–80], seemed to align with the dissociation curve of ITA^2-^.

As the molar ratio of ITA to TOA was 1:1 for the equilibrium experiments in this study, it can be assumed that all ITA was bound to at least one molecule of TOA by ion-pair bonds [112]. To efficiently deplete the organic phase, the ion-pair bonds had to be disrupted. For the sake of simplicity, we chose to perform a pH shift with NaOH [89]. To describe the pH dependence of back-extraction, we subjected a fully loaded organic phase to equal volumes of aqueous phase carrying different NaOH concentrations. As shown in Fig. 2, the depletion of the organic phase followed the dissociation curve of ITA [89].

### Commissioning lab-scale mixer–settlers for reactive extraction and back-extraction

For product removal in ISPR fermentations, we aimed to achieve reactive extraction and back-extraction equilibrium in the mixer–settlers developed in earlier works [101]. Furthermore, for reactive extraction, operating parameters to obtain ITA removal rates matching microbial productivity were to be found. In extended-batch cultivations published previously [5, 37, 38], a fermentation pH of 3.6 and an ITA concentration between 5 g $\hbox {L}^{-1}$ to 40 g $\hbox {L}^{-1}$ resulted in high productivity and yield, thereby delimiting the desired operating window for ISPR and the conditions for mixer–settler operation. For commissioning, we utilized the commonly employed TOA-1-octanol system described in Sect. pH-Dependent extraction of ITA with TOA [56] and continued to use a feed volume phase ratio of $V_{\mathrm {org:aq}}=1:1$. Assuming an ITA concentration of 40 g $\hbox {L}^{-1}$, this phase ratio led to a 1:1.6 molar ratio of ITA to TOA at the maximum ITA concentration of 40 g $\hbox {L}^{-1}$. Thereby, we ensured that extraction was not limited by TOA availability, even if ITA was accumulated in the organic phase or productivity changed.

To assess the yield in mixer–settler operation for reactive extraction relative to the extraction equilibrium, we applied different stirrer speeds ranging from 350 rpm to 500 rpm for extraction of 20 g $\hbox {L}^{-1}$ ITA. As the flow rates determined the residence time and thus the settling time for phase separation, we tested two different flow rates of 5 mL ${\hbox {min}^{-1}}$ and 10 mL ${\hbox {min}^{-1}}$. However, the increased settling time at a flow rate of 5 mL ${\hbox {min}^{-1}}$ did not have a large effect on phase separation. A stirrer speed of 500 rpm was determined sufficient to reach equilibrium in subsequent mixer–settler experiments for both flow rates.

**Fig. 3:**
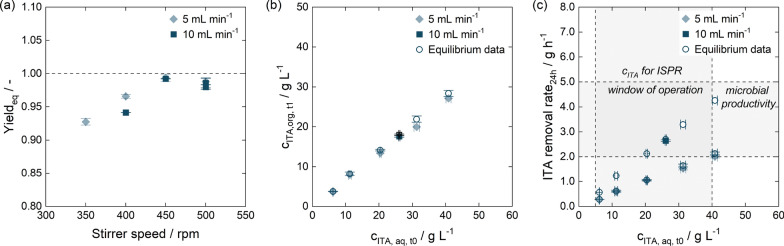
Implementing reactive extraction in a mixer–settler in lab-scale with TOA in 1-octanol. Different feed flow rates of 5 mL ${\hbox {min}^{-1}}$ and 10 mL ${\hbox {min}^{-1}}$ were used. **a** Yield relative to equilibrium depending on stirrer speed when extracting 20 g $\hbox {L}^{-1}$ ITA. **b** Organic phase concentration of ITA depending on initial ITA concentration in the aqueous phase in mixer–settler experiments at a stirrer speed of 500 rpm. Mixer–settler data is compared to equilibrium experiments. **c** Impact of initial ITA concentration on ITA removal rate using data shown in (b) for calculations. The ITA removal rate was calculated assuming one 6 h operation interval per day. The intersection of the desired concentration range for ISPR and the microbial productivity found in literature [37] marks the resulting operating window for ISPR

Extraction efficiency can vary with the loading of the organic phase [68, 75, 113]. To evaluate this effect within the desired operating window for ISPR, we conducted mixer–settler experiments using different starting concentrations of ITA from 5 g $\hbox {L}^{-1}$ to 40 g $\hbox {L}^{-1}$ at an initial pH of 3.6 (Fig. 3b). The ITA concentration in the organic phase increased linearly with the starting concentration in the broth and we derived a constant yield of 67.3 ± 0.4% with 0.5 mol ${\hbox {L}^{-1}}$ TOA in 1-octanol in the ISPR operating window. When comparing equilibrium data to values obtained by mixer–settler experiments, it is noticeable that while a stirrer speed of 500 rpm was sufficient to reach reactive extraction equilibrium at ITA concentrations of 20 g $\hbox {L}^{-1}$, a slight deviation from equilibrium yield was observed for higher ITA concentrations.

With a constant reactive extraction yield, ITA removal rates would decrease linearly as ITA concentrations in the fermenter dropped and would increase if ITA accumulated. The resulting removal rates assuming daily 6 h separation intervals (Sect. Reactive Extraction Performance Evaluation) are depicted for both flow rates in Fig. 3c. The flow rates decided on the amount of broth processed and thus led to different removal rates. Assuming a productivity range from 0.59 g ${\hbox {L}^{-1}}\,\hbox {h}^{-1}$ to 1.4 g ${\hbox {L}^{-1}}\,\hbox {h}^{-1}$ [37] in a fermenter with a filling volume of 3.5 L, reactive extraction in daily 6 h intervals using a flow rate of 5 mL ${\hbox {min}^{-1}}$ would not lead to process conditions within the operating window, but to ITA concentrations exceeding the desired maximum of 40 g $\hbox {L}^{-1}$. Conversely, removal rates at a feed flow rate of 10 mL ${\hbox {min}^{-1}}$ matched fermentation productivity and led to ITA concentrations within the desired range.

Considering the feed phase volume ratio of 1:1 and a yield of 67.3 ± 0.4%, this setup did not produce a concentrated ITA solution in the initial capturing step. A lower feed phase volume ratio of organic to aqueous phase and a higher TOA concentration could increase ITA concentration in the first capturing step. However, as this work was the first proof-of-concept and data on actual productivity in ISPR operation using reactive extraction was not available, we did not pursue optimization of feed phase volume ratios. Instead, we chose to maintain a broad operational window, and reactive extraction not being limited by TOA availability.

**Fig. 4:**
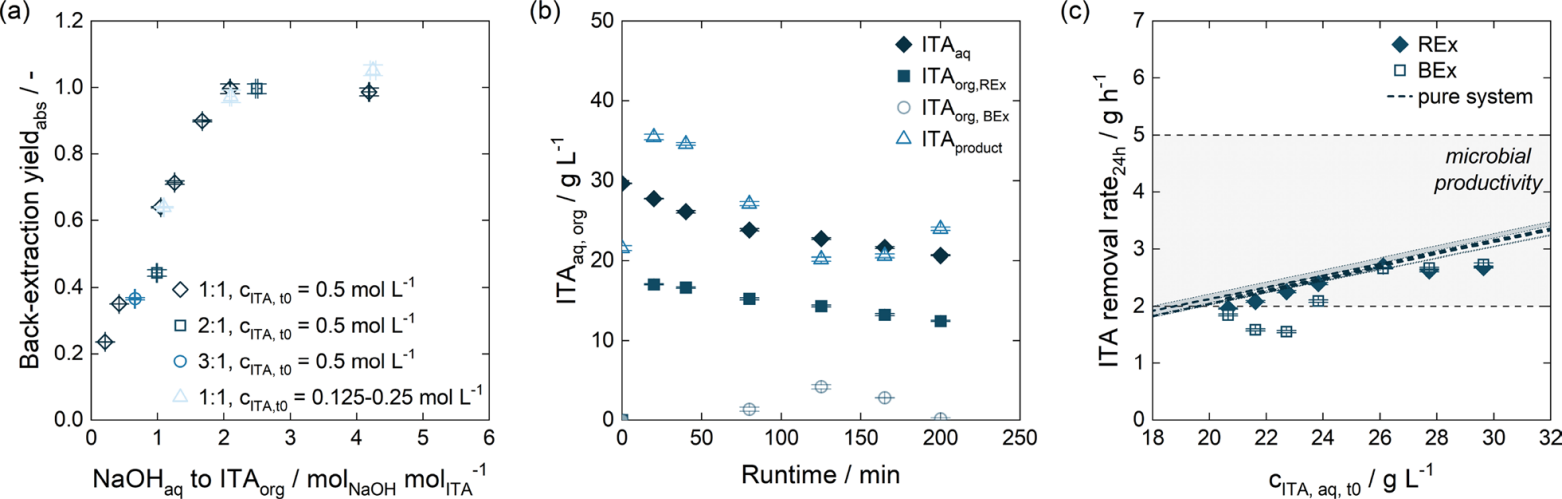
Implementing back-extraction by pH shift in a mixer–settler at lab-scale with TOA in 1-octanol. **a** Back-extraction yield in equilibrium experiments with 0.25 mol ${\hbox {L}^{-1}}$ to 2 mol ${\hbox {L}^{-1}}$ NaOH at different NaOH to ITA ratios for a $V_{\mathrm {org:aq}}=1:1$, $V_{\mathrm {org:aq}}=2:1$ and $V_{\mathrm {org:aq}}=3:1$ feed volume phase ratio. For back-extraction, the organic phase was loaded by reactive extraction at pH 2.0 using an initial ITA concentration of 0.125 mol ${\hbox {L}^{-1}}$, 0.25 mol ${\hbox {L}^{-1}}$ and 0.5 mol ${\hbox {L}^{-1}}$. **b** Continuous reactive extraction and back-extraction with recycling of aqueous and organic phase in mixer–settlers. The organic phase concentrations are determined by back-extraction with 2 mol ${\hbox {L}^{-1}}$ NaOH. **c** Removal rates obtained in continuous reactive extraction and back-extraction with mixer–settlers in comparison with removal rates obtained with single experiments. The expected productivity from literature [37] is indicated by horizontal shading

One way to increase the concentration of ITA in the following process steps is through back-extraction. The equilibrium experiments in Fig. 2 show that a pH shift with NaOH to protonate ITA could effectively deplete the organic phase. If the feed volume phase ratio of organic to aqueous phase is increased in back-extraction and a full depletion of the organic phase is maintained by increasing the NaOH concentration, ITA can be concentrated for further processing. However, high salt concentrations in the aqueous phase might result in a salting-in effect [114, 115] and reduce back-extraction efficiency. Therefore, we depicted the back-extraction yield depending on the NaOH to ITA ratio for equilibrium experiments using different feed volume phase ratio (Fig. 4a). The back-extraction data from Fig.2 with an initial ITA concentration of 60 g $\hbox {L}^{-1}$ in the organic phase was set as a reference. It displays a linear relation of yield and NaOH to ITA ratio until a 2 : 1 ratio is reached and the organic phase is fully depleted. This corresponds well with the conceptual process design for ISPR by reactive extraction published previously [89]. If the feed volume phase ratio was increased to $V_{\mathrm {org:aq}}=2:1$, the back-extraction yields were slightly lower compared to the reference data. Similar effects were observed if the feed volume phase ratio was further increased to $V_{\mathrm {org:aq}}=3:1$, indicating a possible salting-in effect. Alternatively, this slight yield reduction could also be attributed to the phase volume change during back-extraction as discussed in Sect. Reactive Extraction Performance Evaluation and Appendix A.2. To exclude the influence of the ITA concentration in the organic phase, we also investigated back-extraction of 15 g $\hbox {L}^{-1}$ and 30 g $\hbox {L}^{-1}$ ITA in the organic phase with a constant NaOH concentration of 0.5 mol ${\hbox {L}^{-1}}$ at a feed volume phase ratio of $V_{\mathrm {org:aq}}=1:1$. Since the back-extraction yields corresponded with reference data (Fig. 4a), we concluded that the ITA concentration in the organic phase in fact had a negligible influence, and the back-extraction yield in this work was governed predominantly by the ratio of NaOH to ITA.

Based on the data discussed above, we selected a feed volume phase ratio of $V_{\mathrm {org:aq}}=2:1$ for back-extraction as a suitable operating point. This phase ratio allowed sufficient mixing in the mixer–settler unit and slightly increased ITA concentrations in the product phase. As the estimated maximum ITA concentration in the organic phase was at 27.1 ± 0.8g ${\hbox {L}}^{-1}$, this would correspond then to a NaOH concentration of 0.832 mol ${\hbox {L}^{-1}}$ for full depletion of the organic phase. To accommodate for potential ITA accumulation in the organic phase, a NaOH concentration of 1 mol ${\hbox {L}^{-1}}$ was used in future experiments. Similar to reactive extraction, we determined 500 rpm as a suitable stirrer speed (Appendix A.5).

However, after back-extraction in mixer–settlers, a fine turbidity of the organic phase was observed (Appendix A.5). As the organic phase was to be recycled continuously, a turbid organic phase would be used for reactive extraction. In Appendix A.5, we compare reactive extraction yields obtained with a turbid organic phase to yields obtained with a reused organic phase after centrifugation, containing no fine turbidity, and a fresh organic phase. The organic phase containing turbidity performs comparably to both the fresh and centrifuged organic phases at high initial ITA concentrations. At initial ITA concentrations of 6 g $\hbox {L}^{-1}$, the turbid organic phase showed a yield decrease of 7 %. Nonetheless, we decided to recycle the organic phase without prior treatments in subsequent experiments.

To verify the previously determined removal rates, a trial run for extraction and back-extraction was conducted by continuous extraction of 3.5 L ITA solution from a buffer tank at a pH of 3.6 (Fig. 4b,c). Figure 4b illustrates the ITA concentrations in aqueous and organic phases. As more ITA was extracted, the ITA concentration in the buffer tank decreased. The ITA concentration in the organic phase after the reactive extraction showed a slight decline, reflecting the diminishing feed concentration in the buffer tank. Following back-extraction, the ITA content in the organic phase was mainly depleted; however, between 80 min to 165 min, some ITA accumulated in the organic phase. By 200 min, the apparatus reached a steady state, with both the reactive extraction and back-extraction processes functioning equally well. The removal rates for continuous reactive extraction aligned well with removal rates obtained in single mixer–settler experiments, indicated by the dashed line in Fig. 4c. The overall removal rates, which also factored in back-extraction, displayed a slight deviation where ITA accumulated in the organic phase but later recovered to match the efficiency of reactive extraction. This implies successful recycling of the organic phase. The ITA concentration in the product phase was within the range of that in the feed.

Considering further process steps after reactive extraction, an additional concentration step would be necessary. In addition, the high NaOH concentrations within the aqueous phase would require a substantial addition of an acid, such as HCl or H_2_SO_4_ for subsequent crystallization [89]. The corresponding co-salt formation would then reduce the potential crystallization yield [5]. Therefore, in the long run, the pH shift needs to be performed electrochemically for co-salt recycling [59, 64], and the product must be further concentrated by optimizing the phase ratio in reactive extraction and back-extraction.

### Identification of suitable solvent systems

**Fig. 5:**
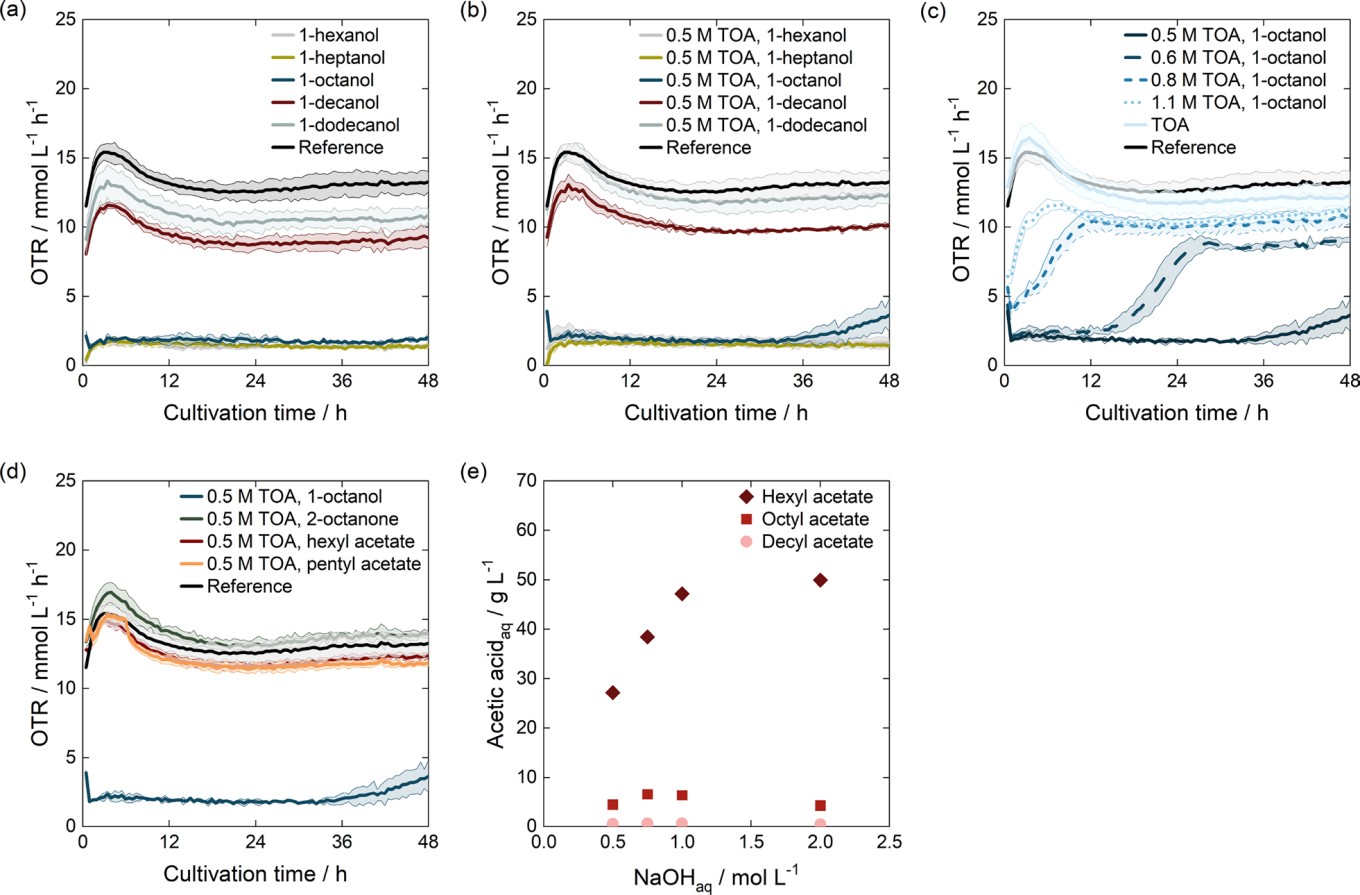
Biocompatibility of different solvents during the production phase. **a** OTR in nitrogen-free medium saturated with diluents. **b** OTR in nitrogen-free medium saturated with 0.5 mol ${\hbox {L}^{-1}}$ TOA in diluents. **c** OTR in nitrogen-free medium saturated with organic solvent using different concentrations of TOA in 1-octanol. **d** OTR in nitrogen-free medium saturated with 0.5 mol ${\hbox {L}^{-1}}$ TOA in different diluents. **e** Stability of ester diluents in back-extraction determined by the formation of acetic acid as hydrolysis product

After implementing mixer–settlers with TOA-1-octanol as the standard system, we assessed the biocompatibility of selected solvents to identify those suitable for ISPR. ITA production by *U. cynodontis* is initiated by nitrogen limitation [37], and biocompatibility tests were, therefore, conducted with nitrogen-limited cells. In the chosen ISPR setup, the cells were retained by a membrane, and no direct contact was to be expected (Fig. 1). Therefore, only molecular toxicity [55, 97] of the cross-dissolved solvent was assessed based on the OTR (Fig. 5). The OTR starting values for each cultivation were slightly different, likely due to minor fluctuations in inoculation density at the beginning of each cultivation.

For an initial evaluation, aliphatic, non-branched C6 to C12 alcohols, frequently used for reactive extraction with TOA [56], were selected and compared to a reference cultivation without organic solvent (Fig. 5a). When the fermentation medium was saturated with 1-decanol or 1-dodecanol, the OTR curves were marginally lower than those of the reference, indicating good biocompatibility. In contrast, a negligible OTR was detected when the medium was saturated with 1-octanol, 1-heptanol, and 1-hexanol. All three solvents show a lower logP and an increased solubility in the aqueous phase. The logP value of diluents is a crucial factor to consider when evaluating solvent toxicity. It decides on the solubility of the solvent in water and its integration into the cell membrane [93, 95, 116]. If the solvent is integrated into the membrane, it can increase or decrease membrane fluidity [117–119], thereby affecting its integrity and leading to a loss of function as a permeable barrier, protein, and reaction matrix, and as an energy transducer [120]. Therefore, a linear correlation between solvent toxicity and logP is often observed for solvents with logP values between 1 and 4 [116]. At logP values above 4, the solubility of the solvent in water is usually too low to cause cell damage [95, 116]. At logP values below 1, however, the solvent is too polar to be integrated into the membrane [95]. In our studies, the critical logP for the biocompatibility of alcohols was between 3.0 and 4.57, corresponding to the logP values of 1-octanol and 1-decanol [121]. It thereby aligned well with literature data [93, 96, 97, 116, 117, 122, 123].

As the experiments in Fig. 5a were conducted without TOA, the impact of adding TOA to the respective solvents is shown in Fig. 5b. The OTR curves for 1-dodecanol, 1-decanol, 1-heptanol, and 1-hexanol were similar. However, the previously flat OTR of 1-octanol showed a slight increase at the end of cultivation. Thus, for further investigations, the medium was saturated with organic phases containing different TOA concentrations in 1-octanol (Fig. 5c). With an increasing TOA concentration, the lag-phase decreased, and the OTR reached higher values. This phenomenon was also observed in literature using TOA in 1-decanol [124]. Since TOA can also extract alcohols, such as phenol [65, 125], it could have acted as a hydrogen acceptor with the hydroxyl group of the alcohol diluent, thereby reducing its solubility in the aqueous phase. Nonetheless, as biocompatibility was still impacted at increased TOA concentrations, 1-octanol was not considered biocompatible. While long-chain alcohols such as 1-decanol and 1-dodecanol seemed more promising, their high viscosities of 11.47 mPa s [126] and 13.72 mPa s [127] caused challenges in phase separation [56](Appendix A.6).

Thus, we investigated the biocompatibility of other diluent groups. Various representatives of alkanes are frequently studied in the literature [56, 128, 129]. Yet, they generally exhibit low extraction efficiencies and tend to form a third phase when used with TOA [102, 124, 130, 131]. Thus, alkanes were not considered in this work. Ketones and esters have also been extensively studied as solvent systems [56]. In contrast to the broad range of alcohols and alkanes explored in the literature, research on ketones primarily focuses on methyl isobutyl ketone [78, 102, 132–134]. Studies on esters are largely confined to butyl acetate [135–137] and ethyl acetate [128]. However, these solvents are all highly water soluble and can affect biocompatibility [55, 96, 97, 122] or might be metabolized by the organism [138, 139]. Thus, we focused on the less water-soluble representatives of these diluent groups. Pentyl acetate, hexyl acetate, and 2-octanone were identified for further analysis. As their viscosities at 25 $^{\circ }\text {C}$ were at 0.86 mPa s, 1.07 mPa s and 1.15 mPa s [140–142], respectively, they were within the viscosity range of standard systems for extraction, such as toluol and butyl acetate [140]. As a result, we anticipated robust phase separation behavior.

Pentyl acetate, hexyl acetate, and 2-octanone have logP values of 2.3, 2.37 and 2.87 [121, 143, 144]. All of these values are below the critical minimum logP value determined for alcohols (Fig. 5a,b) and the diluents are also equally or even better soluble in water than 1-octanol [145–147]. Yet, they showed high biocompatibility (Fig. 5d). These results indicate that while the logP value gives a general indication of solvent toxicity [116, 117], it does not describe specific interactions of the solvent with *U. cynodontis* [95, 96, 148]. While the literature is scarce on the comparison of long-chain alcohols and ketones regarding their biocompatibility, the comparison of ethanol and acetone can illustrate the effect of different functional groups. Ethanol is associated with an inhibition of ATPase [118], a decrease in membrane fluidity [119], and a partial release of phospholipids [149] due to its interaction with the phospholipid head groups [95]. Acetone, on the other hand, has been shown to increase membrane fluidity [119], and is often considered less toxic than ethanol or butanol [149–151], possibly due to different interactions with membrane-bound proteins and phospholipid head groups [118, 119]. Esters and fatty acid methyl esters also have been demonstrated to be generally more biocompatible than alcohols, regardless of logP values in the range of 1-4 [122]. Thus, assuming equal membrane concentrations of 1-octanol, 2-octanone, pentyl acetate, and hexyl acetate, alcoholic diluents could have an additional, target-specific effect [95]. An adaptation of the cells to the solvents by changing membrane composition is also possible [95, 151], but is presumed to occur only to a limited extent, since biocompatibility was tested under growth limitation.

Looking at overall process feasibility, esters, while promising in view of their biocompatibility, were susceptible to basic hydrolysis of the ester bond when equilibrated with NaOH (Fig. 5e). The degree of hydrolysis decreased with increasing ester chain length and the use of short-chain esters as diluents was not advisable for the proposed overall process. Thus, 2-octanone was selected as a suitable solvent for ISPR fermentations. While in this study only 2-octanone was identified as a possible diluent for ISPR, the diluent spectrum can potentially be expanded by branched long-chain alcohols, exploiting their favorable extraction properties [69], and low water solubility at a decreased viscosity. Furthermore, while substituting TOA with TOPO might decrease extraction efficiency [137], it expands the diluent range to long-chain alkanes [132, 152], displaying promising fluid phase properties [56] and higher selectivities toward inorganic anions [133].

**Fig. 6:**
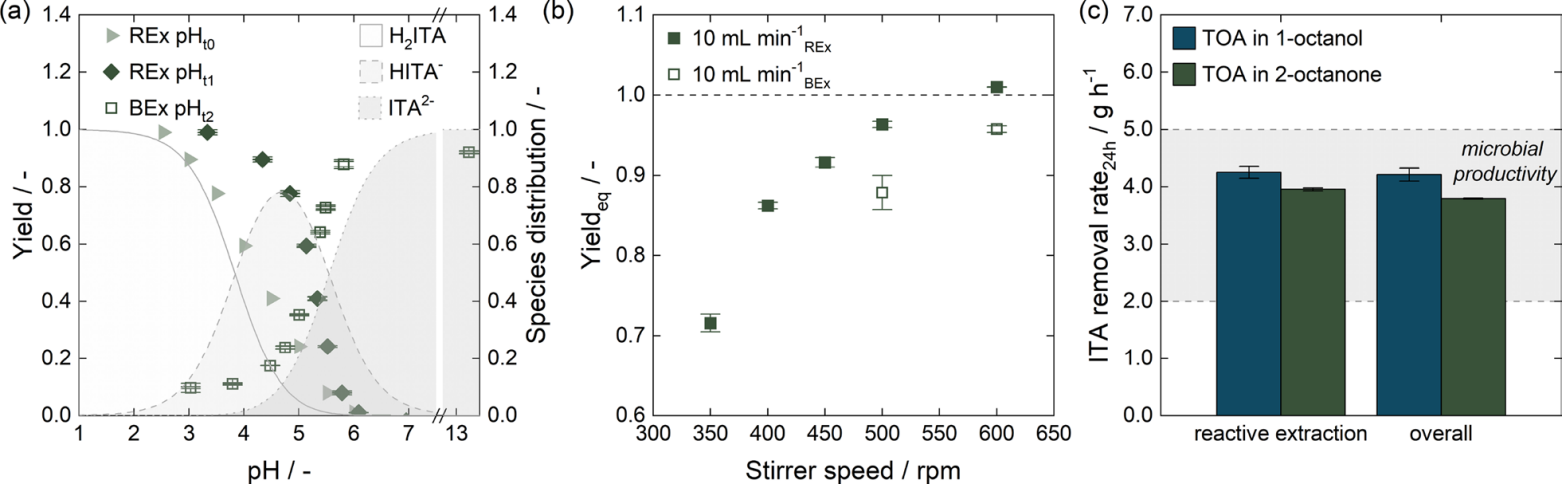
Implementing reactive extraction and back-extraction with 2-octanone as a diluent. **a** Equilibrium data for pH-dependent extraction of ITA by TOA in 2-octanone. The yield is displayed depending on starting pH (REx, ${{\textrm{pH}}_{{t}_{0}}}$) as well as depending on equilibrium pH (REx, ${{\textrm{pH}}_{{t}_{0}}}$), illustrating the pH shift due to ITA extraction. For back-extraction with NaOH, only the equilibrium pH is shown (BEx, ${{\textrm{pH}}_{{t}_{0}}}$). **b** Yield relative to equilibrium in mixer–settlers at different stirring speeds when extracting 40 g $\hbox {L}^{-1}$ ITA in reactive extraction and depleting the organic phase in a subsequent back-extraction. **c** Removal rate in reactive extraction and the overall process with reactive extraction and back-extraction when using TOA in 1-octanol and 2-octanone for extraction of an initial ITA concentration of 40 g $\hbox {L}^{-1}$. The expected microbial productivity from literature [37] is indicated by horizontal shading

To characterize the new solvent system, the pH-dependent extraction yield at equilibrium with 0.5 mol ${\hbox {L}^{-1}}$ TOA in 2-octanone was determined and compared to reactive extraction with TOA in 1-octanol (Fig. 6a). The use of 2-octanone as a diluent resulted in slightly lower extraction efficiencies, particularly at high equilibrium pH values. Nonetheless, at the proposed fermentation pH of 3.6 [37], extraction yields of both systems were equally at around 70%. The pH-dependent equilibrium for back-extraction was slightly shifted at lower pH values, indicating a recovery of partially protonated ITA. Thus, different complex forms might have been present in the two different solvent systems [65, 112, 153].

For implementation in mixer–settlers, different stirrer speeds for reactive and back-extraction were tested (Fig. 6b). In contrast to the TOA in 1-octanol system (Appendices A.4 and A.5), stirrer speeds of 600 rpm were necessary to reach equilibrium for a flow rate of 10 mL ${\hbox {min}^{-1}}$ in reactive extraction and the subsequent back-extraction step. Due to the low viscosity of 2-octanone, phase separation could still be achieved at these stirrer speeds. As TOA in 1-octanol and TOA in 2-octanone showed similar pH-dependent extraction behavior (Figs. 2, 6a), equal ITA concentrations were to be expected in the organic phase, and we continued to use 1 mol ${\hbox {L}^{-1}}$ NaOH for back-extraction at a feed volume phase ratio of $V_{\mathrm {org:aq}}=2:1$.

Figure 6c compares the ITA removal rates for reactive extraction and the overall process with 2-octanone and 1-octanol. The data was obtained by extraction of 40 g $\hbox {L}^{-1}$ ITA at pH 3.6 using a flow rate of 10 mL ${\hbox {min}^{-1}}$. Due to the slightly lower extraction yield (Fig. 6a), the removal rate for reactive extraction with 2-octanone was lower compared to reactive extraction with 1-octanol. Back-extraction, however, was as efficient as with 1-octanol. As a result, the potential overall ITA removal rate in artificial systems decreased marginally from 4.21 ± 0.11 g ${\hbox {h}}^{-1}$ to 3.79 ± 0.01 g ${\hbox {h}}^{-1}$ due to the diluent change.

### Implementing extended-batch fermentations in 5 L scale

**Fig. 7:**
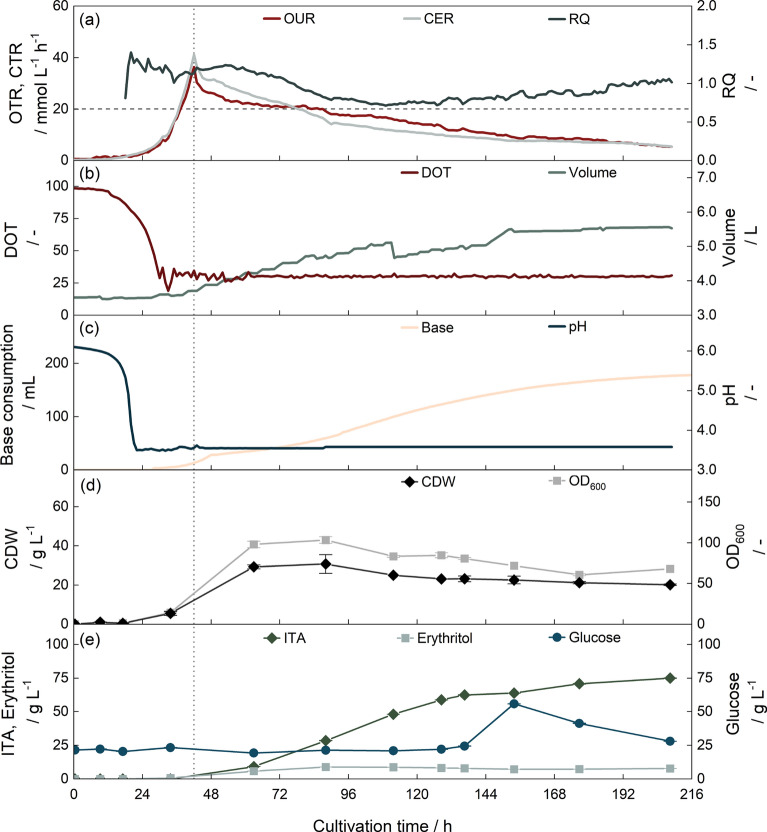
Extended-batch fermentation with *U. cynodontis*. **a** OTR, CTR and RQ. The horizontal dashed line shows the theoretical RQ of 0.67 for ITA production. RQ values are only shown after 24 h. **b** DOT and filling volume. Drops in filling volume result from sampling. **c** pH value and consumption of 10 mol ${\hbox {L}^{-1}}$ NaOH. **d** CDW and OD_600_ **e** Glucose, erythritol and ITA concentration. 20 g $\hbox {L}^{-1}$ glucose were added at the beginning of fermentation, the concentration was controlled by an enzymatic glucose sensor to 20 g $\hbox {L}^{-1}$ afterward. The cultivation was performed at 30 $^{\circ }\text {C}$ with a constant aeration of 3.5 L min$^{-1}$. The DOT was controlled to 30 % by increasing stirrer speed. The dotted horizontal line indicates the transition from growth to production phase after 42 h

After implementing reactive extraction for ITA recovery, we performed an extended-batch cultivation as a reference (Fig. 7). As described in the literature [37, 42], the fermentation started with a growth phase. At 43 h of cultivation, the OTR and CTR peaks marked the end of the growth phase due to nitrogen depletion. Then, the RQ was at values above 1 while predominantly erythritol and some ITA was formed. After 88 h, erythritol production reached its maximum concentration of 8.94 ± 0.03 g $\hbox {L}^{-1}$ and the RQ dropped to values around 0.7, close to the theoretical RQ of 0.67 for ITA production, and the ITA concentration in the fermenter increased. Between 136 h to 153 h of cultivation, the glucose concentration in the fermenter increased from 25 g $\hbox {L}^{-1}$ to 55 g $\hbox {L}^{-1}$ due to challenges in sensor calibration. The RQ, however, remained stable. At the end of fermentation, the RQ increased again, possibly due to weak organic acid stress [154]. The fermentation was terminated after 208 h.

The fermentation KPI obtained were comparable to data from Pastoors et al. (2023) [42] and preceding works from Hosseinpour-Tehrani et al. (2019) [37]. The final titer was at 74.6 ± 0.6 g $\hbox {L}^{-1}$, and thus in the same range as 77.6 g $\hbox {L}^{-1}$[42] and 78.6 g $\hbox {L}^{-1}$ [37] found in literature. The overall substrate yield was at 0.42 ± 0.01 ${\hbox {g}}_{\hbox {ITA}} \hbox {g}_{\hbox {Glc}}^{-1}$. As the extended-batch fermentations by Pastoors et al. (2023) [42] and Hosseinpour-Tehrani et al. (2019) [37], reached yields of 0.38 ${\hbox {g}}_{\hbox {ITA}} \hbox {g}_{\hbox {Glc}}^{-1}$ and 0.45 ${\hbox {g}}_{\hbox {ITA}} \hbox {g}_{\hbox {Glc}}^{-1}$, respectively, the yield in this work is well within the range of literature data. STY was determined to 0.38 ± 0.01 $\hbox {g}_{\hbox {ITA}} \hbox {L}^{-1} \hbox {h}^{-1}$ and is, therefore, slightly lower compared to literature [37, 42]. This corresponds to an increase in the length of the growth phase from approximately 24 h [37] to 41 h due to lower cell densities for inoculation.

**Fig. 8:**
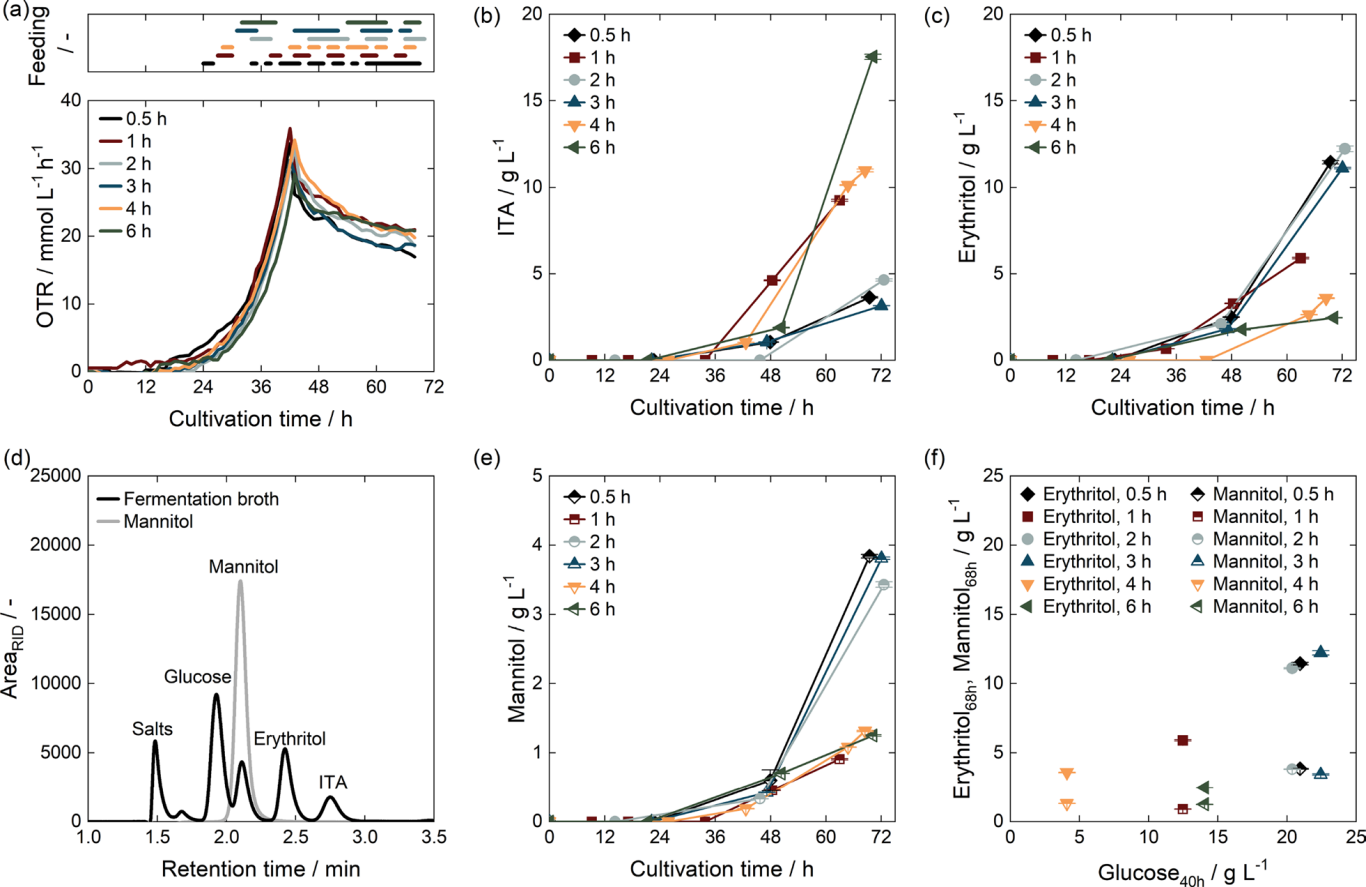
Influence of measuring intervals of the glucose sensor on byproduct formation. Measuring intervals were set to 0.5 h, 1 h, 2 h, 3 h, 4 h and 6 h, and the glucose concentration was controlled between 10 g $\hbox {L}^{-1}$ and 20 g $\hbox {L}^{-1}$. **a** OTR of fermentations. The feeding intervals are marked at the top. Feed flow rates were not considered in this illustration. **b** ITA concentrations. **c** Erythritol concentrations. **d** Identification of mannitol as a byproduct using an HPLC sample after 68 h of fermentation with a 0.5 h feeding interval. **e** Mannitol concentrations. **f** Correlation of erythritol and mannitol formation depending on the glucose concentration at the OTR peak measured by the glucose sensor. Fermentations were performed with an initial volume of 3.5 L at 30 $^{\circ }\text {C}$ with a constant aeration of 3.5 L min$^{-1}$. The DOT was controlled to 30 % by increasing stirrer speed and using an initial stirring rate of 300 rpm. The pH value shifted from 6.0 to 3.6 during the production phase, from where it was controlled by 10 mol ${\hbox {L}^{-1}}$ NaOH. Only the fermentation data for 4 h measuring intervals was derived from fermentation with a controlled pH at 6.0 during the growth phase and a pH shift to 3.6 afterward

Overall, 8.94 $\pm \,$0.03 g $\hbox {L}^{-1}$ erythritol was produced around the time of nitrogen limitation, reducing the yield and prolonging the ITA production (Fig. 7). Prior studies identified the feeding profile as a key factor in byproduct formation [37, 155]. Accordingly, we analyzed the impact on cellular metabolism in short-term fermentations to optimize the transition from growth to production phase. During the first 68 h of the extended-batch cultivation displayed in Fig. 7, glucose concentration was maintained between 20 g $\hbox {L}^{-1}$ to 22 g $\hbox {L}^{-1}$ based on 1 h measuring intervals of the glucose sensor. Since the measuring intervals of the glucose sensor dictated the feeding frequency and the fluctuations in glucose concentration, we assumed a large influence of the control parameters of the glucose sensor on the microbial metabolism. Thus, we varied the length of the measuring intervals for glucose determination and feeding initiation from 0.5 h to 6 h. The extended-batch fermentation data were included for analysis (Fig. 8). The fermentation data using 4 h feeding intervals was derived from a fermentation with a growth phase at pH 6.0 [38] and controlled at a glucose concentration of 10 g $\hbox {L}^{-1}$ rather than the usual 20 g $\hbox {L}^{-1}$. In fermentations with feeding intervals of 0.5 h, 1 h, 2 h, 3 h and 6 h the pH value was allowed to drop freely to pH 3.6 [42] and the glucose concentration was controlled at 20 g $\hbox {L}^{-1}$.

In all cases, the OTR during the growth phase was similar (Fig. 8a), again demonstrating the robust growth of *U. cynodontis* across the pH range 3.6 to 6.0 [37]. After the growth phase, the OTRs changed with the feeding profiles. A glucose measurement frequency of 0.5 h resulted in short, but frequent feeding after initiation of the production phase. After 68 h of cultivation, the OTR reached 17.2 mmolh$^{-1}$L$^{-1}$, leading to an RQ of 1.3. Measurement intervals of 2 h and 3 h produced similar data and also led to RQ values of around 1.3 after 68 h. However, 1 h and 6 h measuring intervals produced higher OTR values after 68 h of 21.3 mmol h$^{-1}$L$^{-1}$ and 23.1 mmol h$^{-1}$L$^{-1}$, respectively. The corresponding RQ values were lower at 1.1 and 0.8, thereby bringing them closer to the theoretical RQ for ITA production, and indicating a suitable glucose measurement frequency. The RQ for 4 h measuring intervals after 68 h of fermentation was at 1.2 with an OTR of 20.3 mmol ${\hbox {L}^{-1}}$. The differences in OTR curves are reflected in ITA and erythritol production (Fig. 8b, c). The 6 h measurement intervals showed the highest ITA and lowest erythritol production, followed by the 1 h and 4 h measurement intervals. In contrast, the 0.5 h, 2 h and 3 h measurement intervals exhibited the opposite trend. Considering the data in Fig. 8 a, b, and c, no clear correlation between glucose measurement interval, ITA production, and erythritol formation could be derived.

During these cultivations, an additional extracellular byproduct was detected by HPLC. Although the occurrence of a second byproduct was already noticed by Hosseinpour-Tehrani (2019) [31], its identity was not clear. Therefore, the literature was analyzed for possible products of Ustilaginaceae sp., and we identified mannitol as an additional byproduct (Appendix A.7). Mannitol co-occurred with erythritol formation (Fig. 8e). Given the osmoprotective properties of mannitol, the actual glucose concentration around the metabolic shift was considered crucial in identifying the mechanisms behind byproduct formation. Varying feeding intervals resulted in different glucose concentrations at the OTR peak. To assess the impact of glucose concentration on metabolic transition to ITA and byproduct formation, we linked the glucose concentration at the OTR peak to the levels of these byproducts at the end of the experiments. It can be concluded from Fig. 8f that byproduct formation increases with increasing glucose concentration during the time of the metabolic shift. However, the fermentation with 6 h feeding intervals deviated from this trend, displaying an extraordinarily low erythritol and mannitol formation (Fig. 8c,e,f). This may be attributed to the fact that the 6 h feed was the only feeding strategy that led to a cessation of feeding around the metabolic shift (Fig. 8a, top), possibly resulting in fewer fluctuations in osmotic pressure. Therefore, low glucose concentrations and a prolonged timepoint for feeding around the OTR peak are effective strategies to reduce erythritol and mannitol formation.

### Implementing a perfusion bioreactor

**Fig. 9:**
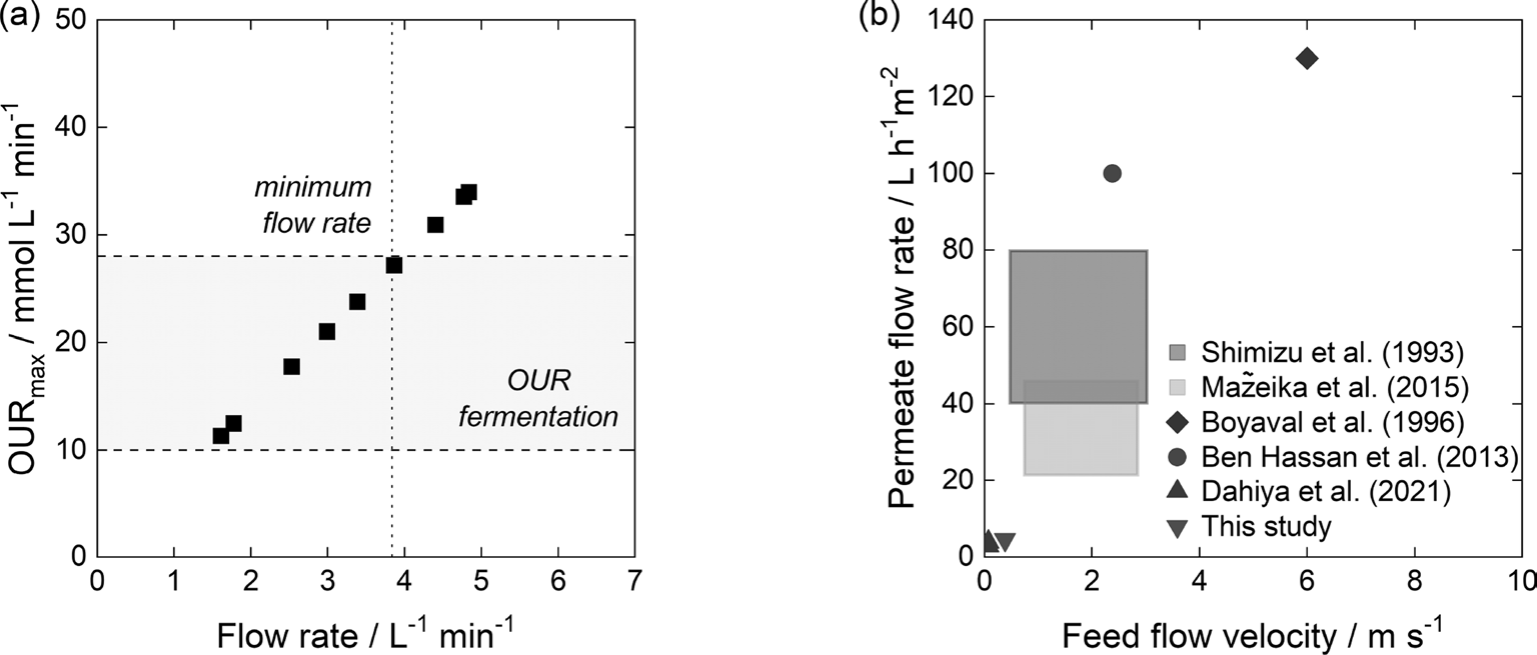
Implementing a membrane for a perfusion-fermenter **a** Correlation of flow rates through the bypass and the maximum OUR for operation without oxygen limitation calculated based on bypass geometry. **b** Permeate flow as a function of feed flow velocity [156–160]. Experiments were conducted with fermentation broth from extended-batch fermentation

For cell retention in ISPR, an external 7-channel hollow fiber ceramic module for cross-flow filtration was connected to the fermenter (ISPR Fermentation in a Perfusion Bioreactor). To determine the operating conditions for the membrane, we used fermentation broth to test different flow rates through the bypass. A maximum flow rate of 4.77 L min$^{-1}$ was reached. At higher flow rates, vibrations potentially caused by entrained gas bubbles from the fermentation increased considerably, making safe operation impossible. Figure 9a links the flow rates to the maximum fermentation OTR possible without oxygen limitation in the bypass, calculated from the residence time, oxygen solubility in water, the DOT, and the bypass volume (Eq. 6). Based on the data obtained in Sect. Implementing Extended-Batch Fermentations in 5 L Scale and the assumption of an OTR equal to OUR (Appendix A.3), we assumed an OUR between 10mmol ${\hbox {h}^{-1}}\,{\hbox {L}^{-1}}$ to 28mmol ${\hbox {h}^{-1}}\,{\hbox {L}^{-1}}$ during the production phase. Consequently, to conduct ISPR fermentations without oxygen limitation in the bypass, the flow rate through the bypass dictates the maximum OUR in fermentation. Thus, the flow rates should be above 3.84 L min$^{-1}$. Given that the maximum flow rate was 4.77 L min$^{-1}$, the flow rate for ISPR operations was set to 4.5 L min$^{-1}$, corresponding to a superficial liquid velocity of 0.379 m s$^{-1}$.

When compared to existing literature [156–161], the feed flow velocity used in this study are low and might not lead to a turbulent flow profile within the membrane channels (Fig. 9b). However, as the permeate flow rate of 10 mL ${\hbox {min}^{-1}}$ was equally low and operating parameters were in a similar range as the study conducted by Dahiya et al. (2021) [160], a stable filtration performance was expected.

### Coupling reactive extraction to fermentation

**Fig. 10:**
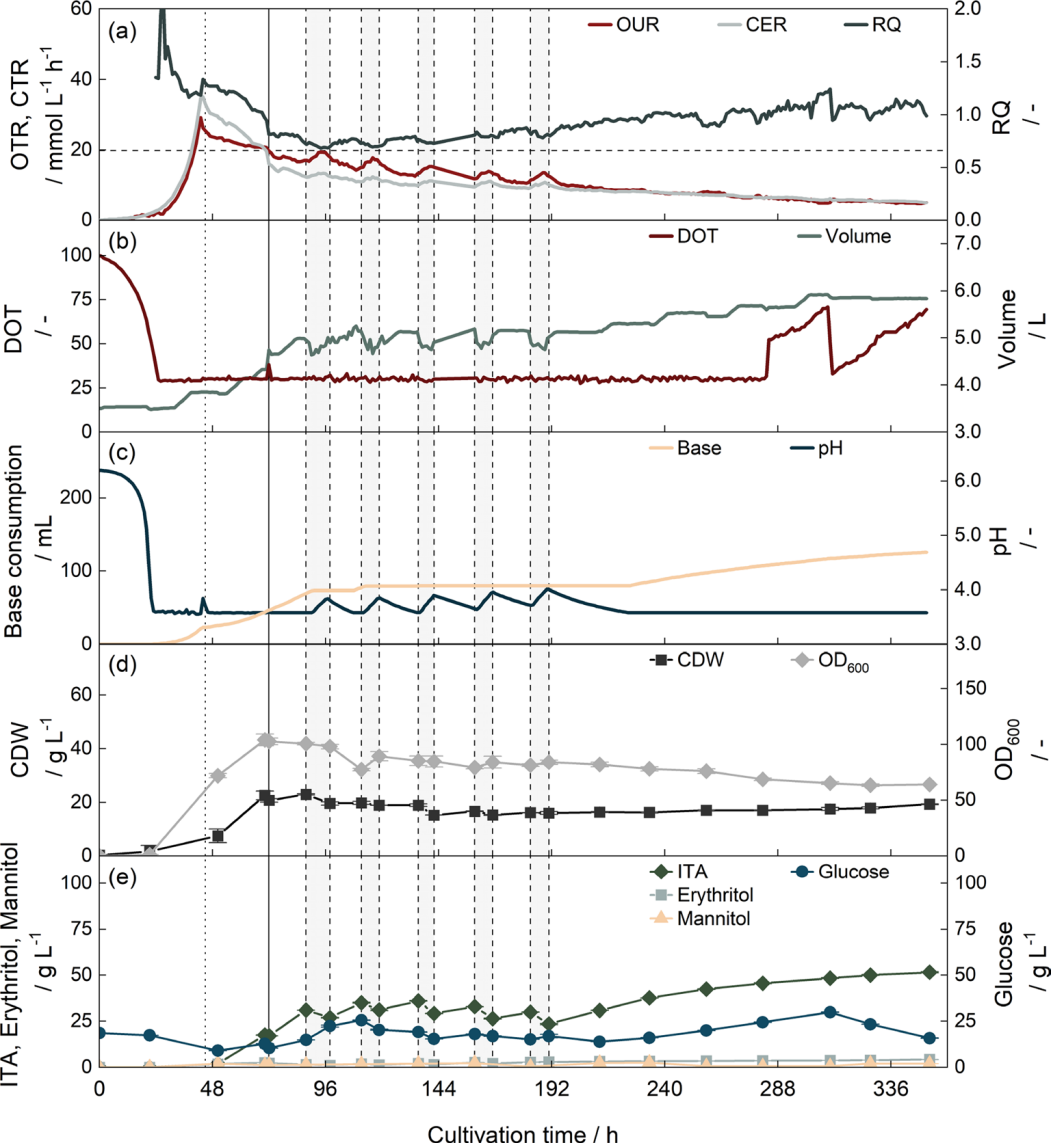
Fermentation results of *U. cynodontis* with ISPR by reactive extraction. **a** OTR, CTR and RQ. The horizontal dashed line shows the theoretical RQ for ITA production. RQ values are only shown after 24 h. **b** DOT and filling volume. Drops in filling volume result from sampling and commissioning of the external membrane loop. **c** pH value and consumption of 10 mol ${\hbox {L}^{-1}}$ NaOH. **d** CDW and OD_600_. **e** Glucose, erythritol and ITA concentration. 20 g $\hbox {L}^{-1}$ glucose was added at the beginning of fermentation, the concentration was controlled by an enzymatic glucose sensor to 20 g $\hbox {L}^{-1}$ afterward. The cultivation was performed at 30 $^{\circ }\text {C}$ with a constant aeration of 3.5 L min$^{-1}$. The DOT was controlled to 30 % by increasing stirrer speed. The dotted horizontal line indicates the transition from growth to production phase. The solid vertical line marks the timepoint of the implementation of the membrane loop. The light gray sections point out the intervals for reactive extraction

In the preceding sections, we implemented mixer–settlers for reactive extraction and back-extraction (Sect. Commissioning lab-scale mixer–settlers for reactive extraction and back-extraction), identified 0.5 mol ${\hbox {L}^{-1}}$ TOA in 2-octanone as a suitable and biocompatible reactive extraction system (Sect. Identification of Suitable Solvent Systems), developed a fermentation protocol (Sect. Implementing Extended-Batch Fermentations in 5 L Scale), and implemented an external membrane (Sect. Implementing a Perfusion Bioreactor). In this chapter, we combine these process steps to an ISPR fermentation. To assure high ITA yield, the fermentation with the lowest erythritol and mannitol formation, due to the improved feeding profile, was continued. For a first proof-of-concept, we decided on five 6 h separation intervals every 24 h prior to the experiment, reflecting the work of Pastoors et al. [42]. Afterward, the fermentation was continued as extended-batch cultivation. In future studies presenting a final process design, an increased number of separation intervals and a final extraction step at the end of cultivation are considered suitable by the authors. The resulting fermentation profile is displayed in Fig. 10.

After the growth phase, the OTR peak at 43 h marks the start of the production phase (Fig. 10a). At 72 h of fermentation, the membrane loop was introduced, causing a slight dilution of broth due to washing water, as indicated by the corresponding increase in reactor volume (Fig. 10b). In addition, the RQ decreased slightly from 1.0 to 0.8, but remained stable afterward until the first separation interval (Fig. 10a). Accordingly, we concluded that the organism tolerated the external membrane.

The drop in RQ coincided with the membrane implementation, but could also be attributed to the improved feeding strategy. The pausing of the feed during the metabolic change from growth to ITA production kept the concentration of erythritol and mannitol both below 5 g $\hbox {L}^{-1}$. The ITA productivity between 63 h and 88 h was increased from 3.76 ± 0.01 g $\hbox {h}^{-1}$ to 4.16 ± 0.05 g $\hbox {h}^{-1}$. A detailed illustration comparing the first 90 h of extended-batch and ISPR fermentations can be found in Appendix A.8.

At 88 h, the first of five 6 h extraction intervals was started. Due to the commissioning of the mixer–settlers, this interval was longer than the following ones. Subsequent separation intervals were initiated after 112 h,136 h, 160 h and 183 h. During each separation interval, both the OTR and CTR slightly increased. Following the findings of Pastoors et al. (2023) [42], this increase in OTR and CTR could be associated with the temporary volume loss when the mixer–settler unit was filled for reactive extraction. However, since the volume change was considered in OTR and CTR calculations, the fermentation broth recycled from the mixer–settler might have been depleted of O_2_ and CO_2_. Thus, the steady state assumed for OTR and CTR calculations (Sect. Fermentation Performance Evaluation, Appendix A.3) was not given anymore. When the broth was led back into fermentation, the amount of O_2_ and CO_2_ transferred into the broth might have been increased due to the dissolution of the gases, rather than their uptake or production.

**Fig. 11:**
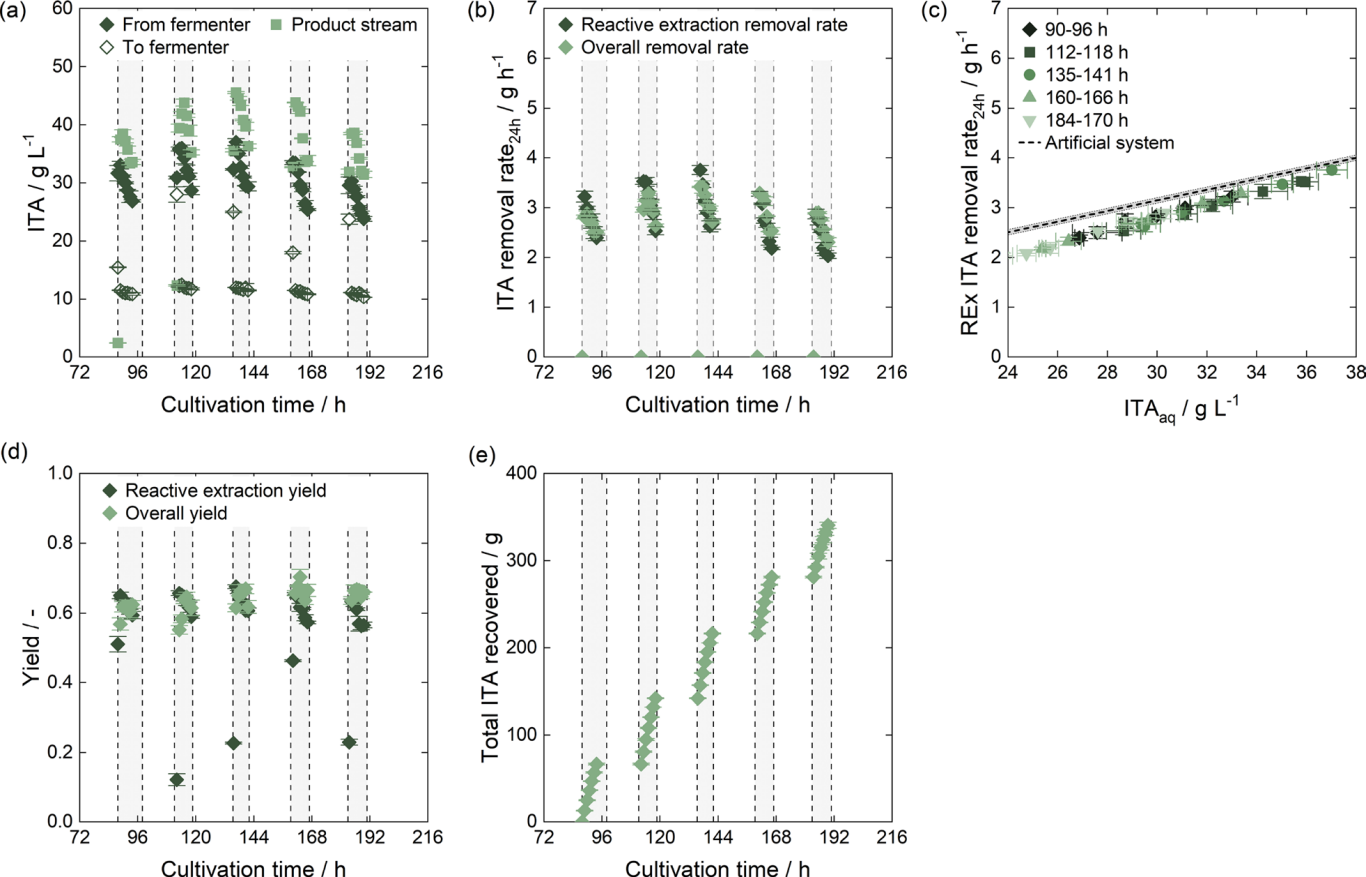
Performance of mixer–settlers in ISPR with TOA in 2-octanone. **a** ITA concentration in the feed from the fermenter, in the depleted medium returning to the fermenter after reactive extraction, and in the product stream. **b** ITA removal rate calculated to 24 h. **c** ITA removal rate to 24 h compared to the removal rate calculated from artificial systems. **d** Yield of reactive extraction and overall yield for both reactive extraction and back-extraction in each separation interval. **e** Total ITA recovered during ISPR fermentation. The light gray sections point out the intervals for reactive extraction

Details on the performance of mixer–settler units can be found in Fig. 11. During each separation interval, the ITA concentration in the broth was successfully reduced by 4 g $\hbox {L}^{-1}$ to 6.5 g $\hbox {L}^{-1}$ (Fig. 11a). The low ITA concentration in the mixer–settler outlet at the start of each interval was assigned to the filling process of the mixer–settler units. For extraction, 2 L of organic phase was used and continuously recycled throughout all five separation intervals. In each interval, a total of 3.6 L of organic phase was brought into contact with 3.6 L of aqueous phase, requiring a simultaneous depletion of the organic phase by back-extraction (Sect. Commissioning lab-scale mixer–settlers for reactive extraction and back-extraction). As the ITA removal rates by reactive extraction and the overall removal rates, combining reactive extraction and subsequent back-extraction, aligned well for all separation intervals, recycling of the organic phase was successful (Fig. 11b). With the depletion of the organic phase, ITA was accumulated in the product stream to concentrations above 30 g $\hbox {L}^{-1}$. The decline of ITA removal rates in each reactive extraction interval was primarily related to the decreasing ITA concentration in the feed phase (Fig. 11a,c).

**Fig. 12:**
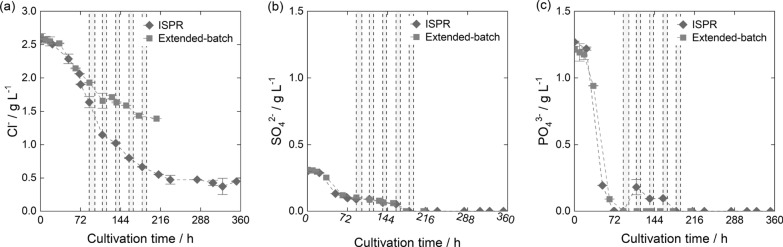
Anion concentration in ISPR and extended-batch cultivations. **a** Cl^-^ **b** SO_4_^2-^ **c** PO_4_^3-^. The light gray sections point out the intervals for reactive extraction

Figure 11c compares the ITA removal rates in ISPR fermentations, depending on the initial ITA concentration, with the data from artificial systems (Fig. 6). While a linear dependence of ITA removal rate and ITA concentration in the feed was observed for both systems, the slope toward higher ITA concentrations was steeper when processing fermentation broth. In addition, the removal rates for fermentation broth were lower, indicating a decrease in extraction yield when transferring from artificial systems to real fermentation broth. This reduced extraction efficiency could have been related to the fine turbidity of the organic phase after back-extraction. The fine turbidity also occurred in artificial systems (Sect. Commissioning lab-scale mixer–settlers for reactive extraction and back-extraction) and led to a slightly reduced extraction yield at low ITA concentrations (Appendix A.5). While we did not quantify the degree of fine turbidity in this work, it is likely that it was enhanced when transferring to real fermentation broth due to the presence of salts and fermentation compounds [56, 162]. In addition, co-extraction of side compounds from fermentation broth could have also led to a decrease in yield. While erythritol and glucose were not extracted (Appendix A3), inorganic anions from the fermentation medium might have been carried over into the organic phase [55, 69, 80, 163]. Figure 12 displays the course of Cl^-^, SO_4_^2-^ and PO_4_^3-^ in ISPR and extended-batch fermentation. While SO_4_^2-^ and PO_4_^3-^ were comparable in both fermentations, the Cl^-^ concentration dropped in the course of the ISPR intervals, indicating a co-extraction. This could not only have led to reduced yields [55], but might have also slowed down extraction kinetics [80]. Therefore, in future studies, the residence time in the mixer–settlers should be assessed using fermentation broth to confirm that the reactive extraction equilibrium is reached. Furthermore, the Cl^-^ ions might have been carried over to the product solution by back-extraction, reducing the available NaOH for ITA recovery and increasing the salt load in the product solution. Considering these effects, future research into reactive extraction systems with increased selectivity toward salts is vital to advance reactive extraction in ISPR fermentations.

Since no acid was used for pH regulation in the fermenter and ITA was removed in its acid form from the broth, the pH of the feed flow to the reactive extraction increased with the progression of separation time. As illustrated by the equilibrium extraction curve in Fig. 6a, this led to a decrease in yield over the separation interval (Fig. 11d). As the organic phase was efficiently depleted, the overall yield for both reactive extraction and back-extraction also mirrored this effect. Each separation interval extracted between 49.5 ± 0.2 g and 79.2 ± 0.5 g ITA in total, until after five separation intervals, 340.7 ± 3.5 g of product were recovered from the fermentation broth (Fig. 11e).

Afterward, the fermentation was continued as an extended-batch cultivation. At 233 h of fermentation, which is similar to the total fermentation time reported in previous ISPR fermentations using adsorption [42], a total of 560.5 ± 4.1 g ITA was produced. This corresponded to an increase of total ITA produced in one extended-batch fermentation by 26 % and highlights the potential of ISPR in regards to reducing bioreactor turnaround time, resulting in a larger overall plant capacity [164]. Furthermore, the STY was also considerably increased from 0.38 ± 0.01 $\hbox {g}_{\hbox {ITA}} \hbox {L}^{-1} \hbox {h}^{-1}$ to 0.46 ± 0.01 $\hbox {g}_{\hbox {ITA}} \hbox {L}^{-1} \hbox {h}^{-1}$, underlining the potential of ISPR to decrease plant footprint [164, 165]. However, the yield, being at 0.44 ${\hbox {g}}_{\hbox {ITA}} \hbox {g}_{\hbox {Glc}}^{-1}$, did not show a strong increase when switching from extended-batch fermentation to ISPR. Similar effects were observed by Pastoors et al. (2023) [42], where the yield from extended-batch to fermentations with ISPR by adsorption was only increased from 0.38 ${\hbox {g}}_{\hbox {ITA}} \hbox {g}_{\hbox {Glc}}^{-1}$ to 0.41 ${\hbox {g}}_{\hbox {ITA}} \hbox {g}_{\hbox {Glc}}^{-1}$. Within the literature, a larger effect was obtained by reducing the amount of nitrogen in the medium and thereby reducing glucose being used for biomass formation [37].

**Fig. 13:**
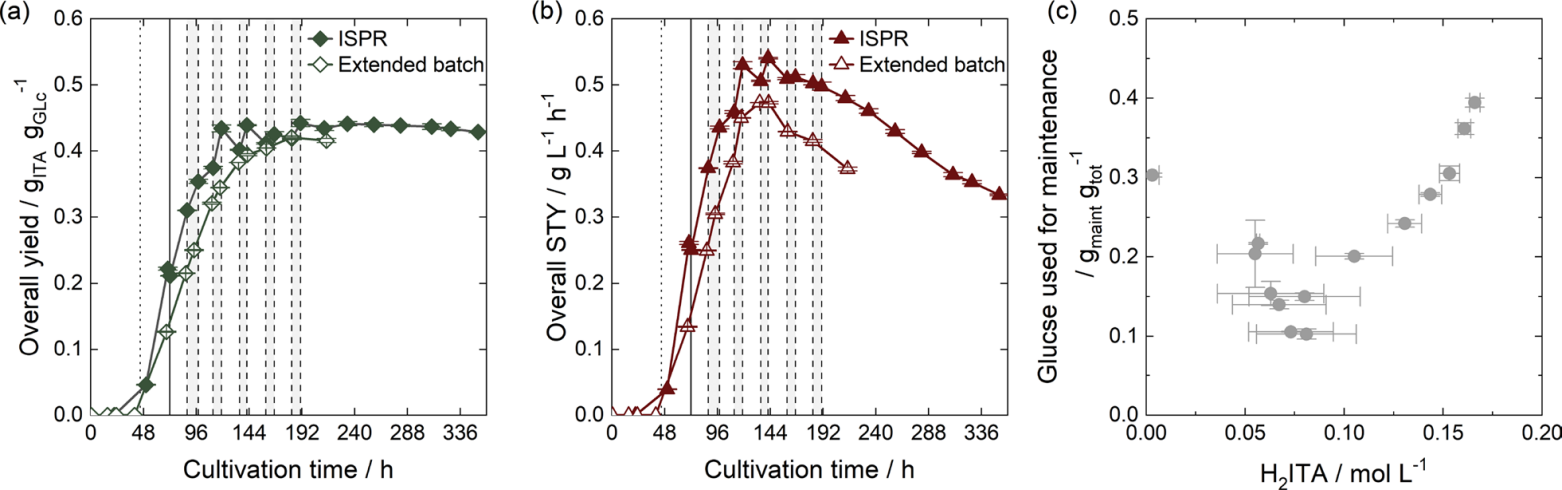
Identification of bottlenecks in ISPR fermentation. **a** Overall yield if the fermentation was terminated at the indicated cultivation time. **b** Overall STY if the fermentation was terminated at the indicated cultivation time. **c** Fraction of glucose used for maintenance depending on H_2_ITA concentration

As yield and STY were anticipated to increase consistently with fermentation time, given that more ITA was produced per CDW and more glucose could be directed toward ITA production, the fermentation was continued until a final fermentation time of 352 h was reached. The fermentation KPI for both 233 h and 352 h are summarized in Table 1 and compared with extended-batch cultivations conducted in this work (Fig. 7) and in the literature [37]. In addition, data from ISPR fermentation using adsorption [42] is included. Contrary to expectations, the yield was slightly lower after 352 h of cultivation. Moreover, the STY was diminished to 0.33 ± 0.01 $\hbox {g}_{\hbox {ITA}} \hbox {L}^{-1} \hbox {h}^{-1}$. An analysis of fermentation KPI in relation to cultivation time (Fig. 13a,b) revealed a near constant yield and a strong decrease in STY after the last reactive extraction interval. Similar effects were observed for the extended-batch fermentation included as a reference. For extended-batch cultivations, this behavior was expected. As the final product concentrations were high, a high product toxicity and thereby strongly reduced productivity and possibly yield occurred at the end of fermentation (Fig. 7). In ISPR fermentations, however, product toxicity was expected to be lower and fermentation KPI should correspondingly be higher. Yet, the increase in yield for ISPR fermentations could primarily be attributed to the decrease of the glucose fraction allocated for biomass formation (Table 2), while the amount of glucose used for maintenance increased from 16.6 ± 0.1% in extended-batch cultivation to 20.8 ± 0.1% in 352 h of ISPR fermentation.

**Table 1:** Fermentation KPI from this work and literature

Protocol^a^	Yield	STY	Titer	Reference
	${\hbox {g}}_{\hbox {ITA}} \hbox {g}_{\hbox {Glc}}^{-1}$	$\hbox {g}_{\hbox {ITA}} \hbox {L}^{-1} \hbox {h}^{-1}$	g $\hbox {L}^{-1}$	
Extended-batch	0.42±0.01	0.38±0.01	74.6±0.6	this work
ISPR_233 h_	0.44±0.00	0.46±0.01	37.6±0.1	this work
ISPR_352 h_	0.43±0.01	0.33±0.01	51.6±0.01	this work
Extended-batch	0.45	0.42	78.6	[37]
Extended-batch	0.38	0.47	77.6	[42]
ISPR	0.41	0.52	55^b^	[42]

^a^ fermentations with 4 g $\hbox {L}^{-1}$ NH_4_Cl and glucose below 20 g $\hbox {L}^{-1}$

^b^ estimated from graph

**Table 2:** Carbon balances of extended-batch and ISPR fermentation

Glucose use	Extended-batch	ISPR_352 h_
	%	%
ITA	57.6±0.4	59.4±0.2
Erythritol	6.7±0.1	3.9±0.1
Mannitol	n.d.^a^	1.2±0.1
CDW	16.2±0.5	11.2±0.1
Maintenance	16.6±0.1	20.8±0.1
Unknown	2.3±0.1	3.4±0.2

$$^{a}$$ not defined

**Fig. 14:**
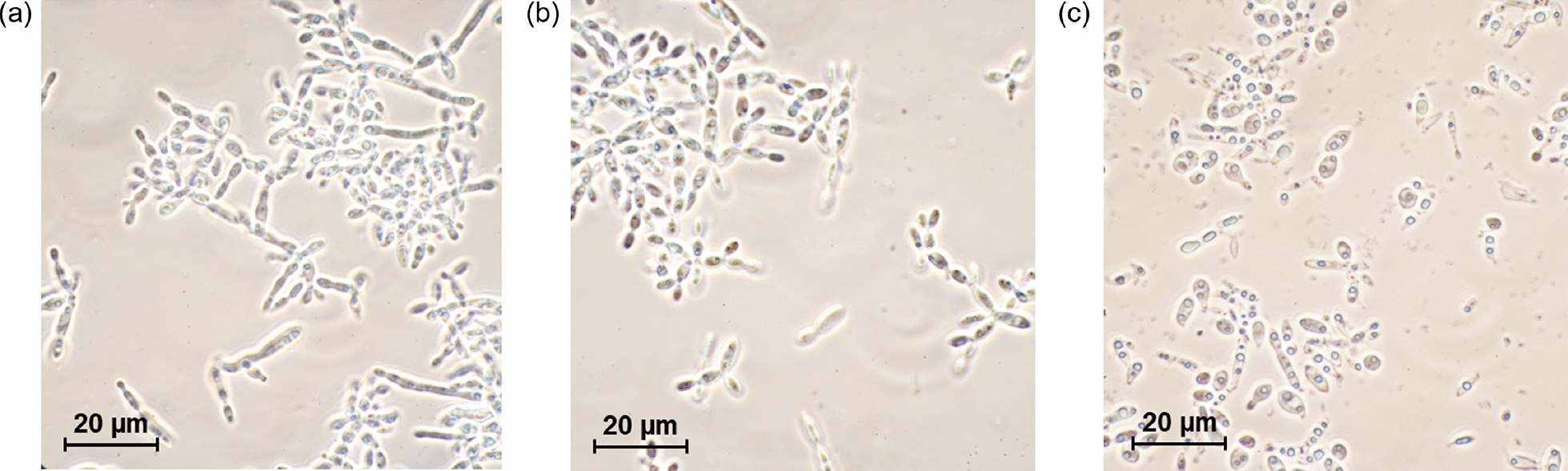
Cell morphology of *U. cynodontis* during the course of ISPR fermentation. **a** Preculture. **b** After 72 h of cultivation before membrane implementation. **c** After 353 h of cultivation

A comparison of the cell morphology from precultivations (Fig. 14a) and before membrane implementation (Fig. 14b) to the cell morphology after 352 h (Fig. 14c) reveals largely damaged, inhomogeneous cells and cell debris at the end of cultivation. This high cell damage and the correspondingly low STY and yield could be attributed to multiple factors. First, the shearing within the external membrane loop in combination with cross-dissolved organic solvent could have enhanced cell stress, leading to a rise in glucose consumption for maintenance and increased cell damage [166]. In addition, while our biocompatibility testing accurately reflected the conditions at the beginning of the production phase, the combined influence of ITA and solvents was not considered. For example, changes in the membrane due to solvent accumulation could have led to a higher susceptibility toward sheer stress [95, 118]. However, as similar fermentation KPIs were obtained by Pastoors et al. (2023) [42] using an internal membrane with lower shear forces for cell retention and activated charcoal for separation, the effect of the external membrane and the cross-dissolved organic solvent was considered small. Second, the co-extraction of anions from the fermentation medium by TOA could have affected ion availability and osmotic pressure in the production phase (Fig. 12). Although this factor should be taken into account in future experiments, growth and production are uncoupled, and while Cl^-^ was extracted, it was not fully depleted. Its influence on cell metabolism compared to extended-batch cultivations is consequently likely minor. However, since SO_4_^2-^ and PO_4_^3-^ were largely depleted, changes in the fermentation could help in increasing production time. Third, as no nitrogen was fed after the growth phase, the increased cell age at the end of fermentation may have contributed to a decrease in productivity during the fermentation course. A high cell age could have also increased the maintenance coefficient at the end of fermentation, and made the cells more susceptible to cross-dissolved organic solvents or changes in the fermentation medium. Fourth, ITA could have also exhibited product toxicity at much lower concentrations than the actual critical concentration of 80 g $\hbox {L}^{-1}$. Straathof (2023) [154] even noted a linear dependence of maintenance and product concentration. Thus, fermentation KPIs could be affected by product toxicity from the start of the production phase. Since the RQ decreased during each separation interval (Fig. 10a), a strong metabolic pressure, even at low product concentrations, seems likely.

Weak organic acid stress is often triggered by the fully protonated acid molecules entering the cells, for example, through aquaglyceroporine channels [167]. Within the cells, they can lower the intracellular pH, bind specifically or non-specifically to various molecules, and induce metabolic changes [154, 168, 169]. Since ITA is possibly transported out of the cell via active transport [106, 170], it is likely that additional ATP was necessary to transport surplus ITA out of the cell. To demonstrate the increased glucose consumption due to weak organic acid stress, the H_2_ITA concentration is plotted as a function of the fraction of glucose being used for maintenance for each sample period (Fig. 13c). While for the overall fermentation the mass balance was closed to 96.6 %, the mass balances in samples directly after the reactive extraction intervals could not be fully closed due to fluctuations in the residual volume in the mixer–settlers. Therefore, for the correlation of glucose being used for maintenance for each sample period, we averaged the data over 24 h intervals from the start of one reactive extraction interval to the start of another. As we also averaged the H_2_ITA concentration over 24 h, the corresponding standard deviations for low amounts of H_2_ITA, where reactive extraction was conducted, were high. Nonetheless, Fig. 13c shows a clear correlation between H_2_ITA concentration and the amount of glucose directed into maintenance. Thus, it is highly likely that weak organic acid stress is a relevant factor in fermentation performance, and a lower ITA concentration could be key to obtaining an ISPR process with a high yield, increased cell viability, and thus longer ISPR periods at a higher STY. In addition, thinking of a final extraction step after termination of fermentation for maximum ITA recovery, a lower ITA concentration could also lead to less ITA being discarded with the fermentation broth (Appendix A.9). This change in operating point could, for example, be achieved by combining an earlier onset of ISPR with an increased length and number of extraction intervals or by switching the reactive extraction system to obtain higher distribution coefficients. It should be noted that a lower ITA concentration could also lead to increased challenges regarding selectivity (Fig. 12), as the ratio of ITA to side compounds would increase.

However, Fig. 13 allows for an additional interpretation. Since particularly high H_2_ITA concentrations were observed at the end of fermentation, the effects of increased cell age and product concentrations may be overlapping, and a combined effect of cell aging and product toxicity is possible. Consequently, to identify the main levers for debottlenecking ISPR cultivations for ITA production with *U. cynodontis*, these effects need to be considered separately in further studies. Only after the identification of ideal operating conditions, the full potential of ISPR can be assessed and weighted against possible disadvantages, such as increased process complexity and apparatus effort [171].

## Conclusion

In this work, we successfully demonstrated the possibility of ISPR by reactive extraction in ITA fermentations using *U.cynodontis* as a promising new production host. We implemented a perfusion bioreactor with an external membrane system coupled to two mixer–settlers for reactive extraction and back-extraction. This approach allowed us to eliminate phase toxicity [63] and challenges in phase separation [61]. Furthermore, we presented the possibility of operating selected reactive extraction systems in dispersion-based apparatuses. Using TOA in 2-octanone as a biocompatible solvent system, we obtained fermentation KPI similar to data published previously by Pastoors et al. (2023) [42] in an adsorption-based process. However, within both ISPR applications, especially the yield was below expectations, suggesting bottlenecks in microbial conversion. In the opinion of the authors, a combination of product toxicity at product concentrations well below the critical concentration of 80 g $\hbox {L}^{-1}$ and increased cell age might limit ITA production [154]. To unleash the full potential of ISPR in ITA fermentations with *U. cynodontis*, the influence of cell age and product concentration on fermentation KPI needs to be investigated further, and improved operating parameters must be found in future studies.

## Data Availability

Data is provided within the manuscript or supplementary information files.

## Abbreviations

CDW: Cell dry weight
CER: Carbon dioxide evolution rate
CTR: Carbon dioxide transfer rate
DOT: Dissolved oxygen tension
DSP: Downstream processing
H_2_ITA: Fully protonated itaconic acid
HITA^-^: Once deprotonated itaconic acid
HPLC: High-performance liquid chromatorgaphy
ISPR: In situ product removal
ITA: Itaconic acid
ITA^2-^: Twice deprotonated itaconic acid
KPI: Key performance indicators
OD_600_: Optical density at 600 nm
OTR: Oxygen transfer rate
OUR: Oxygen uptake rate
RQ: Respiratory quotient
TOA: Trioctylamine
TOPO: Trioctylphosphine oxide

## Symbols

$$\dot{V}$$: Volume flow
*c*: Mass based concentration
*m*: Mass
*R*: Removal rate
*V*: Volume
*Y*: Yield in reactive extraction
*y*: Gas fraction
*N*: Number of samples

## Subscripts

$$\textrm{24h}$$: Averaged to 24 h
$$\textrm{aq}$$: Aqueous phase
$$\textrm{REx}$$: Reactive extraction
$\textrm{BP}$: External membrane loop
$$\textrm{feed}$$: Feed
$\textrm{FS}$: Sample removed from fermenter
$$\textrm{F}$$: Fermenter
$$\textrm{Glc}$$: Glucose
$\textrm{ITA}$: Itaconic acid, all species
$$\textrm{max}$$: Maximum
$$\textrm{NaOH}$$: Sodium hydroxide
$$\textrm{org}$$: Organic phase
$$\textrm{P}$$: Product
$\textrm{RExS}$: Sample removed from reactive extraction
$$\textrm{BEx}$$: Reactive extraction
$\mathrm {t_0}$: Before reactive extraction
$\mathrm {t_1}$: After reactive extraction
$\mathrm {t_2}$: After back-extraction
$\textrm{used}$: Consumed by organism
A: Gas
in: Gas inlet
m: Molar
n: Molar concentration
out: Gas exhaust
